# Expression of Arabidopsis class 1 phytoglobin (*AtPgb1*) delays death and degradation of the root apical meristem during severe PEG-induced water deficit

**DOI:** 10.1093/jxb/erx371

**Published:** 2017-10-20

**Authors:** Mohamed M Mira, Shuanglong Huang, Karuna Kapoor, Cassandra Hammond, Robert D Hill, Claudio Stasolla

**Affiliations:** 1 Department of Botany, Faculty of Science, Tanta University, Tanta, Egypt; 2 Department of Plant Science, University of Manitoba, Winnipeg, Manitoba, Canada

**Keywords:** Arabidopsis roots, auxin, polyethylene glycol, phytoglobin, root meristem, water stress

## Abstract

Maintenance of a functional root is fundamental to plant survival in response to some abiotic stresses, such as water deficit. In this study, we found that overexpression of Arabidopsis class 1 phytoglobin (*AtPgb1*) alleviated the growth retardation of polyethylene glycol (PEG)-induced water stress by reducing programmed cell death (PCD) associated with protein folding in the endoplasmic reticulum (ER). This was in contrast to PEG-stressed roots down-regulating *AtPgb1* that exhibited extensive PCD and reduced expression of several attenuators of ER stress, including *BAX Inhibitor-1* (*BI-1*). The death program experienced by the suppression of *AtPgb1* in stressed roots was mediated by reactive oxygen species (ROS) and ethylene. Suppression of ROS synthesis or ethylene perception reduced PCD and partially restored root growth. The PEG-induced cessation of root growth was preceded by structural changes in the root apical meristem (RAM), including the loss of cell and tissue specification, possibly as a result of alterations in PIN1- and PIN4-mediated auxin accumulation at the root pole. These events were attenuated by the overexpression of *AtPgb1* and aggravated when *AtPgb1* was suppressed. Specifically, suppression of *AtPgb1* compromised the functionality of the *WOX5*-expressing quiescent cells (QCs), leading to the early and premature differentiation of the adjacent columella stem cells and to a rapid reduction in meristem size. The expression and localization of other root domain markers, such as *SCARECROW* (*SCR*), which demarks the endodermis and QCs, and *WEREWOLF* (*WER*), which specifies the lateral root cap, were also most affected in PEG-treated roots with suppressed *AtPgb1*. Collectively, the results demonstrate that *AtPgb1* exercises a protective role in roots exposed to lethal levels of PEG, and suggest a novel function of this gene in ensuring meristem functionality through the retention of cell fate specification.

## Teaser

Retardation of growth induced by water stress is attenuated by the overexpression of *AtPgb1* and aggravated when it is suppressed. Expression of *AtPgb1* is required to delay the death of root tips and to prevent the premature differentiation of meristematic cells.

## Introduction

Among several types of abiotic stress, drought is the most adverse environmental factor affecting plant growth and limiting crop productivity (Westgate and Boyer, 1985). Conditions of water deficit influence both root and shoot behaviour, and plants have developed complex and diverse tolerance and avoidance strategies that are often genotype-specific. While tolerance strategies are usually responses that allow plants to cope with and tolerate the adverse environmental conditions, avoidance strategies allow plants to ‘circumvent’ the stress (Chaves *et al.*, 2002). Both strategies often require extensive reprogramming of gene expression accompanied by metabolic and structural changes (Bohnert and Sheveleva, 1998). A rapid increase in stomatal resistance and alterations in gas exchange in response to growth regulators produced by dehydrating roots are early strategies to limit water loss in photosynthetic tissue (Chaves *et al.*, 2002). These events are often followed by a reduced root growth rate, due to imbalanced cellular water potential compromising elongation, and mechanical impedance from inefficient root penetration in water-depleted soils (Bengough *et al.*, 2011).

In roots, the iterative generation of cells is ensured by the activity of the root apical meristem (RAM), which surrounds a group of mitotically inactive quiescent cells (QCs). The QCs act as an ‘organizing centre’, with the main function to maintain the adjacent stem cells in an undifferentiated state. Stem cells located at the proximal and lateral side of the RAM contribute to the formation of the vascular tissue, endodermis, and cortex, while those situated distally to the QCs are the progenitors of the central domain of the root cap, the columella (Dolan *et al.*, 1993) (see Supplementary Fig. S1A at *JXB* online). This precise and conserved organizational pattern, best exemplified in Arabidopsis, is regulated by a complex genetic network including the transcription factors WUSCHEL RELATED HOMEOBOX 5 (WOX5), SCARECROW (SCR), and WEREWOLF (WER) (Petricka *et al.*, 2012). Alteration of one or more of these factors, expressed in specific root domains (Supplementary Fig. S1B), perturbs the exquisitely regulated regenerative nature of the RAM, resulting in retardation or cessation of growth. A well-documented example is the loss of the undifferentiated state of the columella stem cells following experimental removal of *WOX5* expression (Pi *et al.*, 2015).

Cell fate specification and maintenance in the RAM is influenced by the PIN-mediated transport of auxin which, through a basipetal flow, accumulates in the root tip. Among the several PIN members, the most well characterized in relation to root behaviour are PIN1, which with its basal localization on the plasma membrane creates a root tip-directed auxin flow (Vieten *et al.*, 2005), and PIN4, which is implicated in the short-range redistribution of auxin within the RAM (Friml *et al.*, 2002) (see Supplementary Fig. S1A). Pharmacological treatments or genetic manipulations that alter either the flow of auxin or the establishment of auxin maxima at the tip disrupt the patterning and functionality of the RAM, hence compromising root growth.

Perturbations in RAM function occur under conditions of water deficit, with a mild stress causing the premature differentiation of the RAM (Ji *et al.*, 2014), while a more severe stress triggers the death of the meristematic cells (Duan *et al.*, 2010). Autophagic programmed cell death (PCD) of the meristematic cells has been implicated in root-tip death of several species, including pea and maize exposed to severe stress conditions (Subbaiah and Sachs, 2003). Widespread death was also observed in Arabidopsis RAMs grown in a polyethylene glycol (PEG) environment generating water potentials less than –1.40 MPa (Duan *et al.*, 2010). The death program was triggered by an imbalance between folded and unfolded proteins in the endoplasmic reticulum (ER), which represents a common cellular stress (Williams *et al.*, 2014). In both animals and plants, adverse environmental conditions contribute to the accumulation of misfolded proteins in the secretory pathway which, if not mitigated by the activation of the highly conserved unfolded protein response (UPR), leads to apoptosis and PCD (Boyce and Yuan, 2006). Attenuators of the ER-stress mediated death program, such as BAX inhibitor-1 (BI-1), reduce cellular death (Watanabe and Lam, 2006).

Retention of meristem function and protection of meristematic cells from death under suboptimal environmental conditions have been postulated to be an avoidance strategy modulated by phytoglobins (Pgbs) (Mira *et al.*, 2017). Phytoglobins, formerly known as non-symbiotic plant hemoglobins (Hill *et al.*, 2016), are heme-containing proteins that act as scavengers of nitric oxide (NO) and suppressors of the death program (Huang *et al.*, 2014). Expressed in the root tip (Dordas *et al.*, 2003; Zhao *et al.*, 2008) and induced under conditions of biotic and abiotic stress, Pgbs exercise a protective role during hypoxic (low-oxygen) conditions (Hill, 2012). Overexpression of *Pgb*s enhanced survival of hypoxic roots in Arabidopsis (Hunt *et al.*, 2002), alfalfa (Dordas *et al.*, 2003), and maize (Mira *et al.*, 2016*b*). In maize, the overexpression of *Pgb*s contributes to the retention of a functional meristem by repressing the death program triggered by oxygen deprivation (Mira *et al.*, 2016*b*). Based on these premises, we speculated that the protective function of Pgbs represents a strategy adopted by plants to cope with diverse types of stress, including severe water deficit. This notion was tested in the present study by evaluating the effects of altered expression of the Arabidopsis class I *Pgb* (*AtPgb1*) on the behaviour and structure of the RAM in roots experiencing severe PEG-induced water stress that caused death of wild-type root cells.

## Material and methods

### Plant material

*Arabidopsis thaliana* (Columbia) plants overexpressing (35S:Pgb1) or suppressing (Pgb1-RNAi) phytoglobin1 were those characterized by Hebelstrup *et al.* (2006). The β-glucuronidase (GUS) or green fluorescent protein (GFP) reporter lines crossed with the 35S:Pgb1 and Pgb1-RNAi lines included PIN1-GFP (Fernandez-Marcos *et al.*, 2011), WOX5:GFP and SCR:GFP (Zhang *et al.*, 2015), WER:GFP (Rodriguez *et al.*, 2015), PIN4:GUS and DR5:GUS (Li *et al.*, 2015), CYCB1;2:GUS (Zhang *et al.*, 2010*a*), and CYCB1;3:GUS and CYCA1;2:GUS (Bulankova *et al.*, 2013). Crossing was conducted (as in Mira *et al.*, 2016*c*) using the 35S:Pgb1 and Pgb1-RNAi lines as pollen donors. Arabidopsis seeds were sterilized in 70% ethanol containing 0.5% Triton X-100 for 15 min followed by 95% ethanol for 15 min (Mira *et al.*, 2016*c*) The seeds were then plated on agar germination medium [Murashige and Skoog (MS) salt with vitamins, 2.5% sucrose (w/v) and 0.8 % agar (w/v), pH 5.7], incubated for 24 h at 4 °C in the dark, and then transferred to a growth cabinet (22 °C, 16 h light/8 h dark). The PEG-containing medium was prepared as previously described by Duan *et al.* (2010). Briefly, the desired concentration of PEG-8000 was dissolved in full-strength MS salts containing 2.5% sucrose (pH 5.8). The solution was autoclaved [121 °C, 20 psi (~138 kPa)] for 10 min, cooled to room temperature, and poured on solid medium (full-strength MS salts, 2.5% sucrose and 0.8% agar). The plates were incubated at room temperature for 48 h to allow the PEG solution to infiltrate the solid medium. Arabidopsis seedlings (4 d-old) were placed on freshly prepared PEG medium and the growth of the root was measured at 0, 2, 4, and 6 d.

### Chemical treatments

The inhibitor of ethylene perception 1-methylcyclopropene (1-MCP) was administered at a concentration of 1 ppm (Takahashi *et al.*, 2015). The seedlings were incubated with 1-MCP in a sealed container during the PEG treatment. Diphenyleneiodonium chloride (DPI), an inhibitor of NADPH oxidase (Huang *et al.*, 2014), and sodium 4-phenylbutyrate (PBA), an attenuator of ER-stress (Welch and Brown, 1996), were applied at concentrations of 40 μM and 1 mM respectively. The solutions (10 μl) were dispensed every other day directly on the PEG-treated roots. The ethylene-releasing agent Ethephon (ET) (Zhang *et al.*, 2010*b*) was applied at a concentration of 10 μM.

### Microscopy

Roots were stained with propodium iodide (10 µg ml^–1^) for 5 min, rinsed with distilled water, mounted with 10% glycerol, and visualized using a confocal Zeiss LSM 700 microscope. Signals for propodium iodide and GFP were visualized using a 570–670 nm filter and 500–550 nm filter, respectively. Image J (https://imagej.nih.gov/ij/index.html) was used to measure the GFP signal and the area of expression. The size of the meristem (calculated as described by González-García *et al.*, 2011) included the region from the QCs to cells that had twice the length of the immediately preceding ones. Starch granules in the columella cells were visualized using the Lugol’s staining method (Hong *et al.*, 2015). Roots were immersed in Lugol’s solution (Sigma) for 30 s, cleared with a solution containing chloral hydrate, glycerol, and water in a 8:3:1 ratio, and imaged by differential interference contrast microscopy. For GUS staining, roots were immersed in acetone for 10 min, rinsed twice in 100 mM sodium phosphate buffer (pH 7.2), infiltrated for 15 min with 100 mM sodium phosphate buffer (pH 7.2) containing 1 mg ml^–1^ 5-bromo-4-chloro-3-indolyl β-glucuronide, 10 mM EDTA, 10 mM potassium ferrocyanide, and 0.1% Triton X-100, and incubated at 37 °C for 5 h (Mira *et al.*, 2016*c*).

### ROS and PCD detection

A terminal deoxynucleotidyl transferase-mediated dUTP nick-end labeling (TUNEL) assay was performed to detect PCD using an In Situ Cell Death Detection Kit (Roche), according to the manufacturer’s instructions. Cell death was also estimated by staining the roots with Trypan blue and propidium iodide as described previously (Leite *et al.*, 1999; Ning *et al.*, 2002). Reactive oxygen species were localized using dihydroethidium, as reported by Mira *et al.* (2016*b*).

### DNA isolation and electrophoresis

For DNA fragmentation analyses, 100 mg of the root tips were frozen and ground in liquid nitrogen. The samples were digested using cellulase (1%) for 2 h. DNA extraction was performed using the Apop-ladder EX™ DNA fragmentation assay kit (Clontech Laboratories Inc.) according to the manufacturer’s protocol. The samples were separated on 2% agarose gel.

### Ethylene measurements

Ethylene measurements on Arabidopsis seedlings were performed as previously described (Mira *et al.*, 2016*b*). Briefly, 200 mg fresh weight of seedlings was incubated in a sealed 3-ml syringe for 3 h in the dark at 22 °C. The gas (1 ml) accumulated in the headspace was analysed with a Bruker 450-GC Gas Chromatograph. Data analysis was carried out using the Bruker Compass Data analysis 3.0 software. All experiments were performed in triplicate.

### Gene expression studies

Extraction of RNA from roots was performed using TRI Reagent (Mira *et al.*, 2016*c*). The total RNA was treated with DNase I (recombinant, RNase-free, Roche). A High-Capacity cDNA Reverse Transcription Kit (Applied Biosystems) was used for cDNA synthesis. Quantitative RT-PCR was performed as previously described (Mira *et al.*, 2016*c*). All primers used for gene expression studies are listed in Supplementary Table S1. The relative gene expression level was analysed using the 2^−∆∆CT^ method (Livak and Schmittgen, 2001) with *UbQ10* as the reference gene.

### Statistical analysis

Data were analysed by one-way ANOVA using the SPSS program (IBM Corp. Released 2010. IBM SPSS Statistics for Windows, Version 19.0. Armonk, NY: IBM Corp.). Treatments means were compared using Duncan’s test (α=0.05) to differentiate the significance of differences between various parameters.

## Results

### Modulation of *AtPgb1* expression influences root sensitivity to water stress

**Fig. 1.**
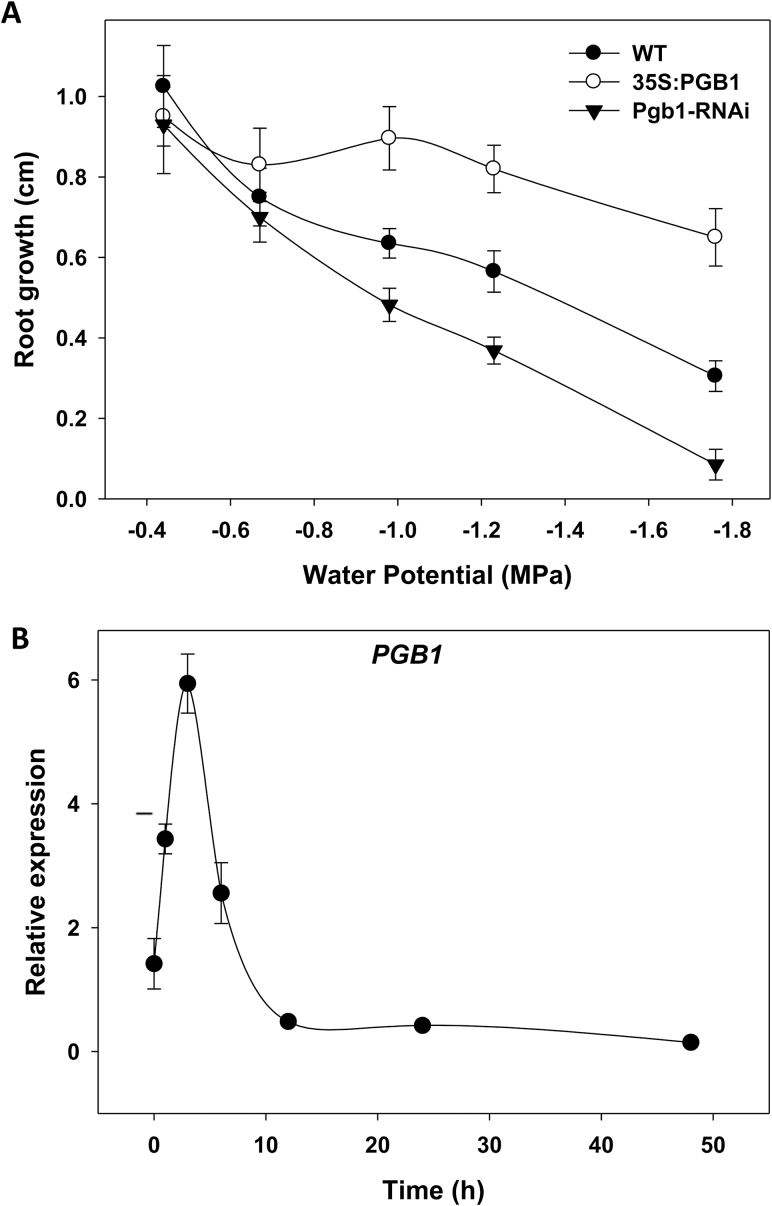
(A) Effects of decreasing water potential on root growth of a wild-type (WT) line and lines overexpressing (35S:Pgb1) or down-regulating (Pgb1-RNAi) *AtPgb1*. Arabidopsis seedlings were 4 d old when placed on PEG-containing medium, and root length was measured after 6 d. Values are means ±SE of three biological replicates. (B) Time-course expression of *AtPgb1*in wild-type roots grown in 40% PEG (–1.76 MPa). Expression values are means ±SE of three biological replicates.

Drought stress in 4-d-old Arabidopsis seedlings was induced by infusing the medium with different concentrations of PEG-8000 corresponding to water potential values ranging from –0.42 MPa (0% PEG) to –1.76 MPa (40% PEG) (see Supplementary Fig. S2). Autoclaving the PEG solution for only 10 min had no significant effects on the water potential, as similar values were obtained when the PEG solution was filter-sterilized (Supplementary Fig. S2). In wild-type (WT) seedlings, elongation of the primary root was inhibited with increasing levels of PEG, which lowered the water potential of the medium (Fig. 1A). The inhibitory effect of PEG was more pronounced in Arabidopsis plants suppressing *AtPgb1* (line Pgb1-RNAi), where root elongation was almost completely inhibited with 40% PEG (–1.76MPa). This was in contrast to plants overexpressing *AtPgb1* (line 35S:Pgb1), which showed reduced sensitivity to water deficit and substantial root growth at the highest PEG levels (Fig. 1A and Supplementary Fig. S3). As the most significant differences in root growth patterns among lines were observed with 40% PEG (–1.76 MPa), this concentration was used for all the subsequent experiments. A time-course of root elongation of the three lines at –1.76 MPa is shown in Supplementary Fig. S4.

The expression of *AtPgb1* was monitored in WT roots cultured at –1.76 MPa (Fig. 1B). Water stress induced a rapid increase in the transcript levels of *AtPgb1*, which reached a peak after 3 h in PEG before declining. An increase in *AtPgb1* expression, albeit less pronounced, was also observed on lower levels of PEG (see Supplementary Fig. S5).

### *AtPgb1* suppresses ER stress-mediated PCD in PEG-treated roots

**Fig. 2.**
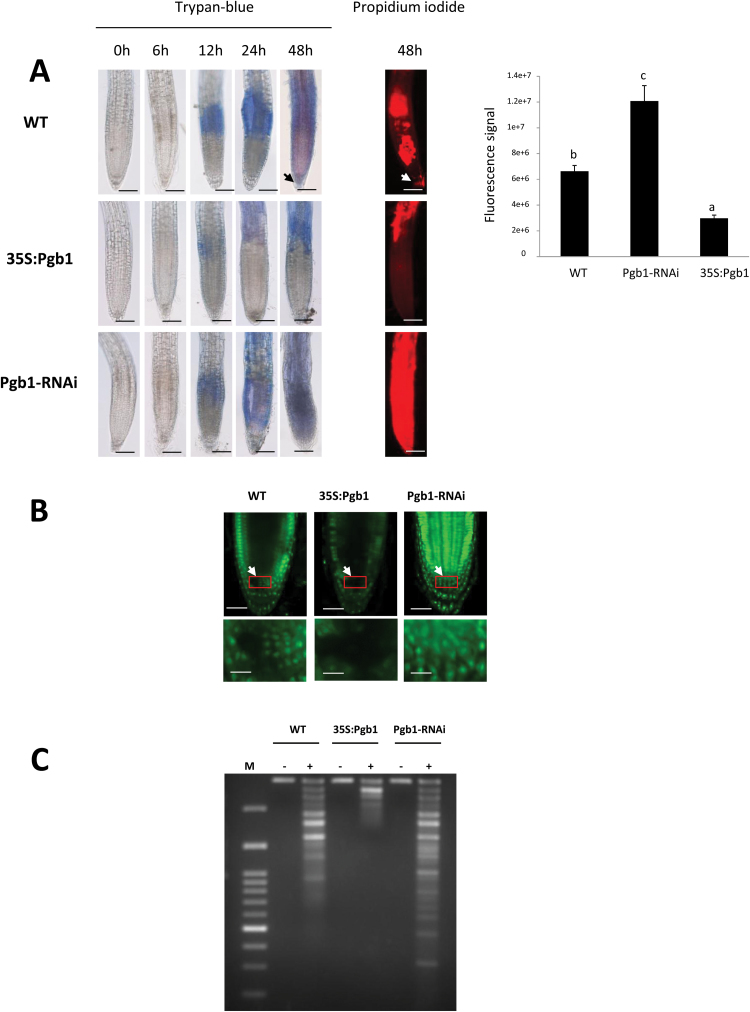
Programmed cell death (PCD) in roots exposed to 40% PEG (–1.76 MPa). (A) Trypan blue and propidium iodide staining of roots of the wild-type (WT) and lines overexpressing (35S:Pgb1) or down-regulating (Pgb1-RNAi) *AtPgb1*. Roots were grown on 40% PEG and stained at different time points (as indicated). The arrow indicates staining in the root tip of WT roots. The graph indicates the fluorescence signal (in pixels) of propidium iodide within the root apical tip (1 mm). Scale bars are 100 μm. Values are means ±SE of three biological replicates. Different letters on the graph indicate statistically significant differences (*P*<0.05). (B) Terminal deoxynucleotidyl transferase dUTP nick-and labelling (TUNEL) staining in the root tips after 48 h in 40% PEG. Arrows indicate the centre of the root apical meristem (RAM). The bottom panels are higher magnification images of the RAMs enclosed in the red boxes. Scale bars are 50 μm (upper panels) and 20 μm (lower panels). (C) Fragmentation of DNA extracted from roots of different lines grown for 48 h in the presence (+) or absence (–) of PEG. M, molecular weight marker.

Many types of stress, including water deficit, limit plant growth by inducing death in root cells (Subbaiah and Sachs, 2003; Duan *et al.*, 2010). Cell death progression in Arabidopsis roots subjected to water stress was examined using Trypan blue, a dye that can only penetrate damaged plasma-membranes and stain dead cells (Huang *et al.*, 2014). In WT roots, substantial Trypan staining was initially apparent after 12 h of water deficit within the upper domain of the roots encompassing the elongation and differentiation zones (Fig. 2A). Following prolonged PEG treatment, the signal progressed apically along the root profile, reaching the RAM at 48 h (arrow in Fig. 2A). Overexpression of *AtPgb1* (line 35S:Pgb1) delayed cell death, which was excluded from the root tip, while roots suppressing *AtPgb1* (line Pgb1-RNAi) were almost completely stained by Trypan blue after only 24 h of PEG treatment (Fig. 2A). The staining pattern of Trypan blue at 48 h in PEG was also confirmed using propidium iodide, another stain unable to penetrate live cells (Ning *et al.*, 2002) (Fig. 2A).

As documented previously (Duan *et al.*, 2010), the death program induced in root cells by water deficit is a type of PCD. This was apparent in the present study after 48 h in PEG. TUNEL staining was detected in several nuclei along the profile of WT roots, and in close proximity to the RAM (arrow in Fig. 2B). This was in contrast to roots up-regulating *AtPgb1* where the death program was restricted to very few cells, and excluded the RAM. Many TUNEL-positive nuclei were detected in Pgb1-RNAi roots (Fig. 2B). This trend was also confirmed by the DNA profile, which revealed some laddering in WT and *AtPgb1*-suppressing roots, but not in roots where the level of *AtPgb1* was increased (Fig. 2C). Detection of only a moderate DNA ladder, as observed in our study, has been ascribed to the fact that during water stress not all of the root cells undergo PCD (Gunawardena *et al.*, 2004).

**Fig. 3.**
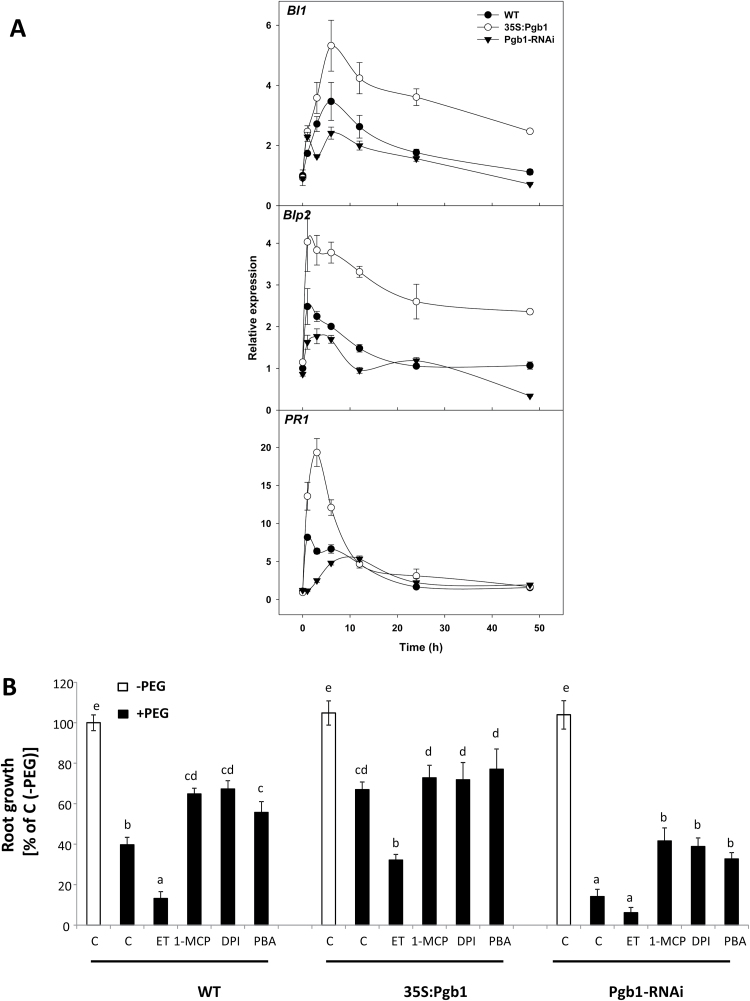
Endoplasmic reticulum (ER) stress-induced PCD in water-stressed roots. (A) Expression levels of *BAX Inhibitor-1* (*BI-1*), *Luminal binding protein 2* (*BiP2*), and *Pathogenesis-related protein 1* (*PR1*) in roots treated with 40% PEG (–1.76 MPa) for the wildtype (WT) and lines overexpressing (35S:Pgb1) or down-regulating (Pgb1-RNAi) *AtPgb1*. Values are normalized to the WT at 0 h (set at 1), and are means ±SE of three biological replicates. (B) Effects of 4-phenyl butyric acid (PBA), 1-methylcyclopropene (1-MCP), ethephon (ET), and diphenyleneiodonium (DPI) on root growth. Values are expressed as percentages relative to the respective control in the absence of PEG (C, –PEG) (set at 100%), and are means ±SE of three biological replicates, each consisting of 20 roots. Different letters indicate statistically significant differences (*P*<0.05). When applied, PEG was added at a concentration of 40% (–1.76 MPa).

The ER participates in PCD by sensing and relaying cellular apoptotic signals through the balance between folded and unfolded proteins (Boyce and Yuan, 2006). Over-accumulation of misfolded or unfolded proteins is perceived as a form of stress which, if not mitigated by the activation of the unfolded protein response (UPR), escalates to PCD (Boyce and Yuan, 2006). The expression levels of the attenuator of the ER stress-mediated PCD, *BAX Inhibitor-1* (*BI-1*) (Watanabe and Lam, 2008), and the markers of UPR activation, *Luminal binding protein 2* (*BiP2*) and *Pathogenesis-related protein 1* (*PR1*) (Duan *et al.*, 2010), were highly induced, especially within the first 10 h, in water-stressed roots overexpressing *AtPgb1* (Fig. 3A). This was in contrast to roots suppressing *AtPgb1* which, relative to the WT, exhibited the lowest expression levels of the three genes (Fig. 3A). These transcriptional studies suggest that expression of *AtPgb1* attenuates ER stress.

To further elucidate the role of *AtPgb1* in alleviating ER stress-mediated PCD we used 4-phenyl butyric acid (PBA), a stabilizer of protein confirmation and attenuator of ER stress (Welch and Brown, 1996). Applications of PBA to the *AtPgb1*-suppressing roots partially relieved the PEG-growth inhibition (Fig. 3B), possibly by suppressing PCD (see Supplementary Fig. S6).

Collectively, these findings confirm the notion that severe water deficit induces PCD in root apices (Duan *et al.*, 2010), and suggest that modulation of *AtPgb1* expression influences root growth by regulating ER stress-mediated PCD of meristematic cells. Relative to the WT, growth inhibition and PCD were induced in roots where *AtPgb1* was down-regulated and mitigated in those where *AtPgb1* was up-regulated.

### *AtPgb1* regulation of death in PEG-stressed roots is mediated by ethylene and ROS

We have previously shown that the protective role of Pgbs on hypoxic maize root cells is exercised through the suppression of ethylene and ROS, elicitors of PCD (Mira *et al.*, 2016*a*, 2016*b*). To establish if similar mechanisms operate during drought, we conducted studies on ethylene and ROS in PEG-treated roots. Relative to the WT, ethylene accumulation (measured in whole seedlings due to limitations in harvesting sufficient root tissue) was more pronounced in water-stressed seedlings suppressing *AtPgb1* and less pronounced in those where the level of *AtPgb1* was up-regulated (Table 1). These changes were most likely the result of transcriptional changes of key ethylene biosynthetic genes (see Supplementary Fig. S7).

**Table 1.** Ethylene levels (nmol g^–1^ FW h^–1^) measured in Arabidopsis seedlings of the wild-type (WT) and lines overexpressing (35S:Pgb1) or down-regulating (Pgb1-RNAi) *AtPgb1* cultured for 24 h in 40% PEG (–1.76 MPa). Values are means ±SE of three biological replicates. Different letters indicate statistically significant differences (*P*<0.05)

	**–PEG**	**+PEG**
WT	0.135 ± 0.016^a^	0.422 ± 0.048^c^
35S:Pgb1	0.137 ± 0.014^a^	0.292 ± 0.022^b^
Pgb1-RNAi	0.134 ± 0.028^a^	0.703 ± 0.037^d^

**Fig. 4.**
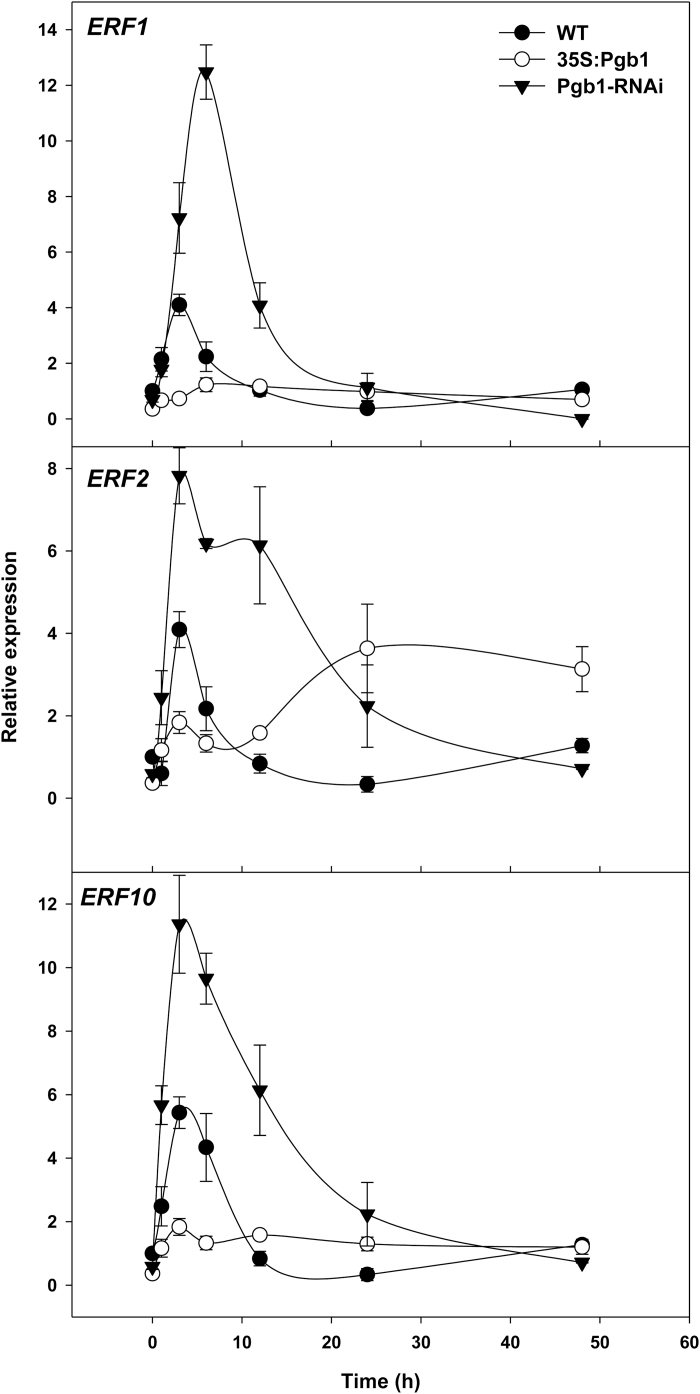
Expression levels of the *Ethylene Responsive Factor 1* (*ERF1*), *ERF2*, and *ERF10* in roots treated with 40% PEG (–1.76 MPa) of the the wild-type (WT) and lines overexpressing (35S:Pgb1) or down-regulating (Pgb1-RNAi) *AtPgb1*. Values are normalized to the WT at 0 h (set at 1), and are means ±SE of three biological replicates.

Ethylene response, estimated by the expression of *ETHYLENE RESPONSIVE FACTOR1* (*ERF1*), *ERF2*, and *ERF10*, was also influenced by Pgbs. Relative to water-stressed WT roots, the expression of the three genes during the first hours in PEG was generally induced in roots down-regulating *AtPgb1* and suppressed in roots up-regulating *AtPgb1* (Fig. 4).

The requirement of ethylene for the AtPgb1 phenotype was further established pharmacologically with 1-MCP, an ethylene-perception inhibitor (Takahashi *et al.*, 2015), partially relieving the growth retardation of the *AtPgb1*-suppressing roots, and ET, an ethylene-releasing agent (Zhang *et al.*, 2010*b*) limiting growth of the *AtPgb1* overexpressing roots (Fig. 3B). These effects were ascribed to changes in PCD patterns (see Supplementary Fig. S6).

**Fig. 5.**
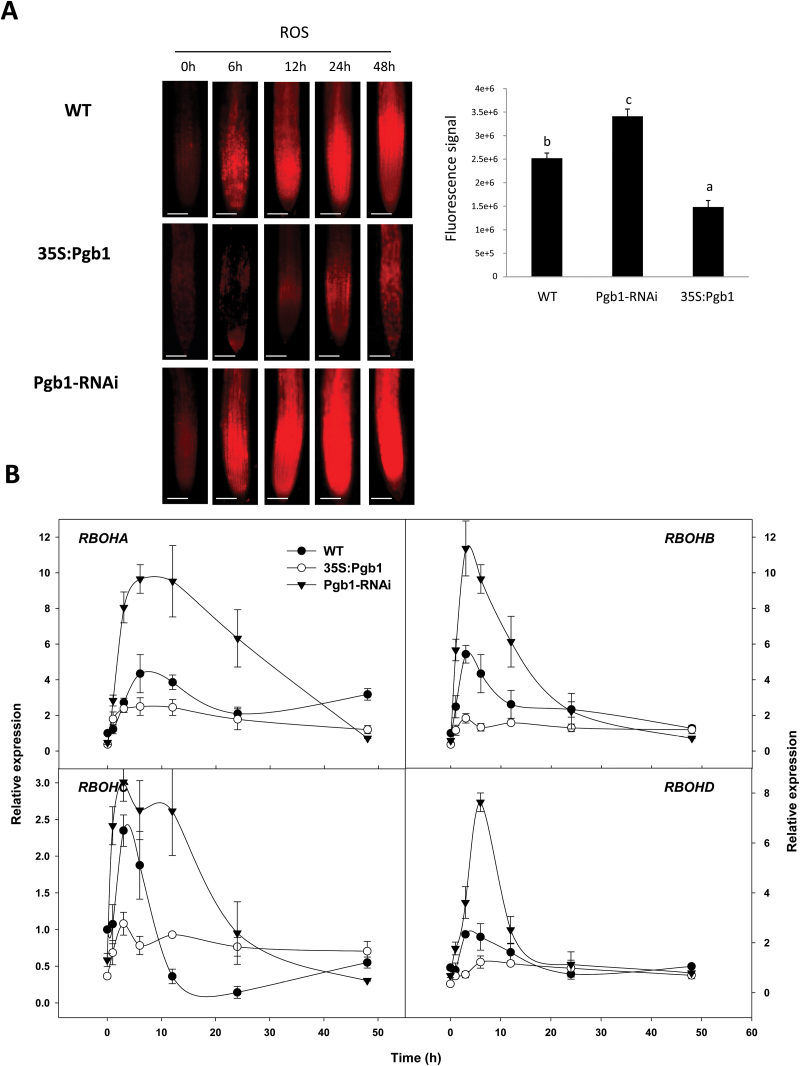
Phytoglobin effects on reactive oxygen species (ROS) during water stress. (A) Localization of ROS in the wild-type (WT) and lines overexpressing (35S:Pgb1) or down-regulating (Pgb1-RNAi) *AtPgb1*. Roots were grown on 40% PEG (–1.76 MPa) and stained at different time points (as indicated). Scale bars are 100 μm. The graph indicates the fluorescence signal (in pixels) within the root apical tip (1mm) at 48 h. Values are means ±SE of three biological replicates. Different letters on the graph indicate statistically significant differences (*P*<0.05). (B) Expression levels of the Arabidopsis *respiratory burst oxidases* (*RBOHA–D*) in roots treated with 40% PEG. Values are normalized to the WT at 0 h (set at 1), and are means ±SE of three biological replicates.

Together with ethylene, ROS act as downstream components in the *AtPgb* regulation of hypoxic responses (Mira *et al.*, 2016*a*, 2016*b*). During the PEG treatment, accumulation of ROS was more pronounced in the *AtPgb1*-suppressing roots and less in those overexpressing *AtPgb1* (Fig. 5A). Production of ROS in plant cells is mainly regulated by the activity of NADPH oxidases, a family of enzymes induced under suboptimal environmental conditions (Sagi and Fluhr, 2006). In mammals, NADPH oxidase is composed of several components, including the glycosylated transmembrane protein gp9^1*phox*^ (Torres and Dangl, 2005). Expression of the four Arabidopsis respiratory burst oxidases (*RBOHA–D*), homologs to gp91^phox^ and reliable indicators of ROS generation (Lin *et al.*, 2009), increased markedly in roots suppressing *AtPgb1*, peaking rapidly after only a few hours on PEG-medium (Fig. 5B). Relative to WT roots that exhibited a more moderate *RBOH* induction, roots overexpressing *AtPgb1* had a generally lower expression of the same genes with the exception of *RBHOC* after 12 h of PEG treatment (Fig. 5B). The involvement of NADPH oxidase-generated ROS production in the cessation of root growth observed in the *AtPgb1*-suppressing line was demonstrated with diphenyleneiodonium (DPI), an inhibitor of NADPH-oxidase (Huang *et al.*, 2014). Inclusion of DPI partially restored growth in water-stressed roots suppressing *AtPgb1* (Fig. 3B), and this was associated with a decrease in PCD (see Supplementary Fig. S6).

In addition to demonstrating the involvement of both ethylene and ROS in the *AtPgb1* regulation of root growth under PEG stress, these results, in the context of previous findings (Mira *et al.*, 2016*a*, 2016*c*), indicate that *AtPgb1* expression may act as a strategy to protect plant cells from death and to sustain growth under severe stress conditions through reduction of ROS and ethylene levels within stressed cells.

### *AtPgb1* is required for RAM maintenance and functionality in water-stressed roots

**Fig. 6.**
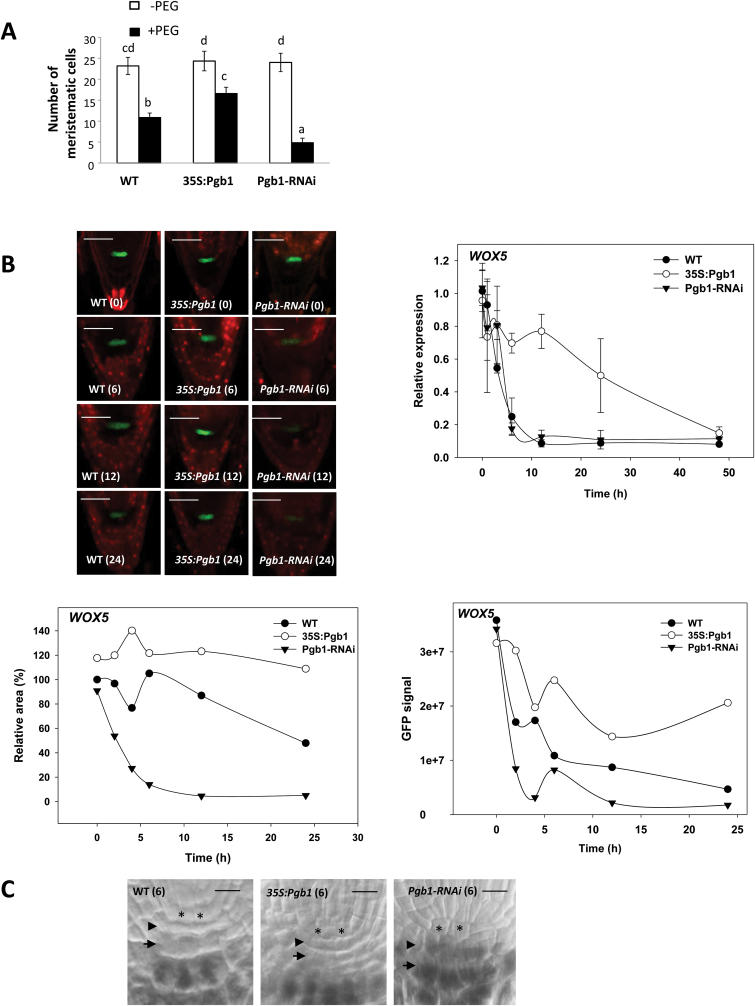
Analysis of meristem size and WOX5 localization and expression in water-stressed roots. (A) Number of meristematic cells in roots of the wild-type (WT) and lines overexpressing (35S:Pgb1) or down-regulating (Pgb1-RNAi) *AtPgb1* grown for 48 h in the absence or presence of 40% PEG (–1.76M Pa). Values are means ±SE of three biological replicates, each consisting of at least 10 sections. Different letters indicate statistically significant differences (*P*<0.05). (B) Confocal images of WOX5:GFP marking the quiescent cells (QCs). Numbers in brackets on the images indicate hours of treatment in PEG. Scale bars are 50 μm. The graphs show the relative area of *WOX5* expression (normalized to the WT at 0 h, which was set at 100%), the intensity of the GFP signal (in pixels), and the relative abundance of the *WOX5* transcripts (normalized to the WT at 0 h, which was set at 1) in roots of the different lines grown in the presence of 40% PEG. (C) Meristems of roots cultured for 6 h in 40% PEG stained for starch granules (black precipitates) with Lugol’s solution. Quiescent cells (*), columella stem cells (arrowhead), and the most immediate columella stem cell derivatives (arrows) are shown. Scale bars are 15 μm.

To assess if the death program in water-stressed roots is preceded by alterations in RAM patterning, the structure of the root tip in the different lines was analysed. Water stress reduced the number of root meristematic cells and this effect was attenuated in plants overexpressing *AtPgb1* and was more apparent in those suppressing *AtPgb1* (Fig. 6A). To establish if the reduction in meristem size was accompanied by malfunctions of the meristematic cells and to identify the exact meristematic domains influenced by *AtPgb1*, we crossed lines with altered levels of *AtPgb1* with reporter lines (WOX5:GFP, SCR:GFP, and WER:GFP) that demark specific root domains (see Supplementary Fig. S1B). The GFP signal for WOX5, a reliable marker of the quiescent cells (QCs) in the RAM (Pi *et al.*, 2015), declined slowly in water-stressed WT roots (Fig. 6B). In roots suppressing *AtPgb1*, the fluorescence almost completely disappeared after a few hours in PEG. This rapid decline in the GFP signal was accompanied by a reduction in *WOX5* expression area and transcripts. PEG-treated roots overexpressing *AtPgb1* retained the WOX5 signal and exhibited the highest levels of *WOX5* transcripts (Fig. 6B). The function of the WOX5-expressing QCs is to act as an ‘organizing centre’ conferring ‘stem state’ to the surrounding cells, and the loss of this state, resulting from *WOX5* suppression, compromises root growth (Pi *et al.*, 2015). To assess if the rapid loss of the WOX5 signal in PEG-treated roots suppressing *AtPgb1* causes premature and terminal differentiation of the distal columella stem cells, we examined the differentiation status of the columella by staining for starch granules with Lugol’s stain. This stain is routinely used to distinguish between the starchless columella stem cells (and their immediate derivatives) from the starch-containing differentiated columella cells (González-García *et al.*, 2011). After 6 h in PEG, both WT and 35S:Pgb1 roots exhibited two starchless layers of cells below the QCs (asterisks in Fig. 6C): the columella stem cells (arrowheads) and their most immediate derivatives (arrows). This was in contrast to roots suppressing *AtPgb1*, which showed early signs of differentiation in the distal columella stem cells.

**Fig. 7.**
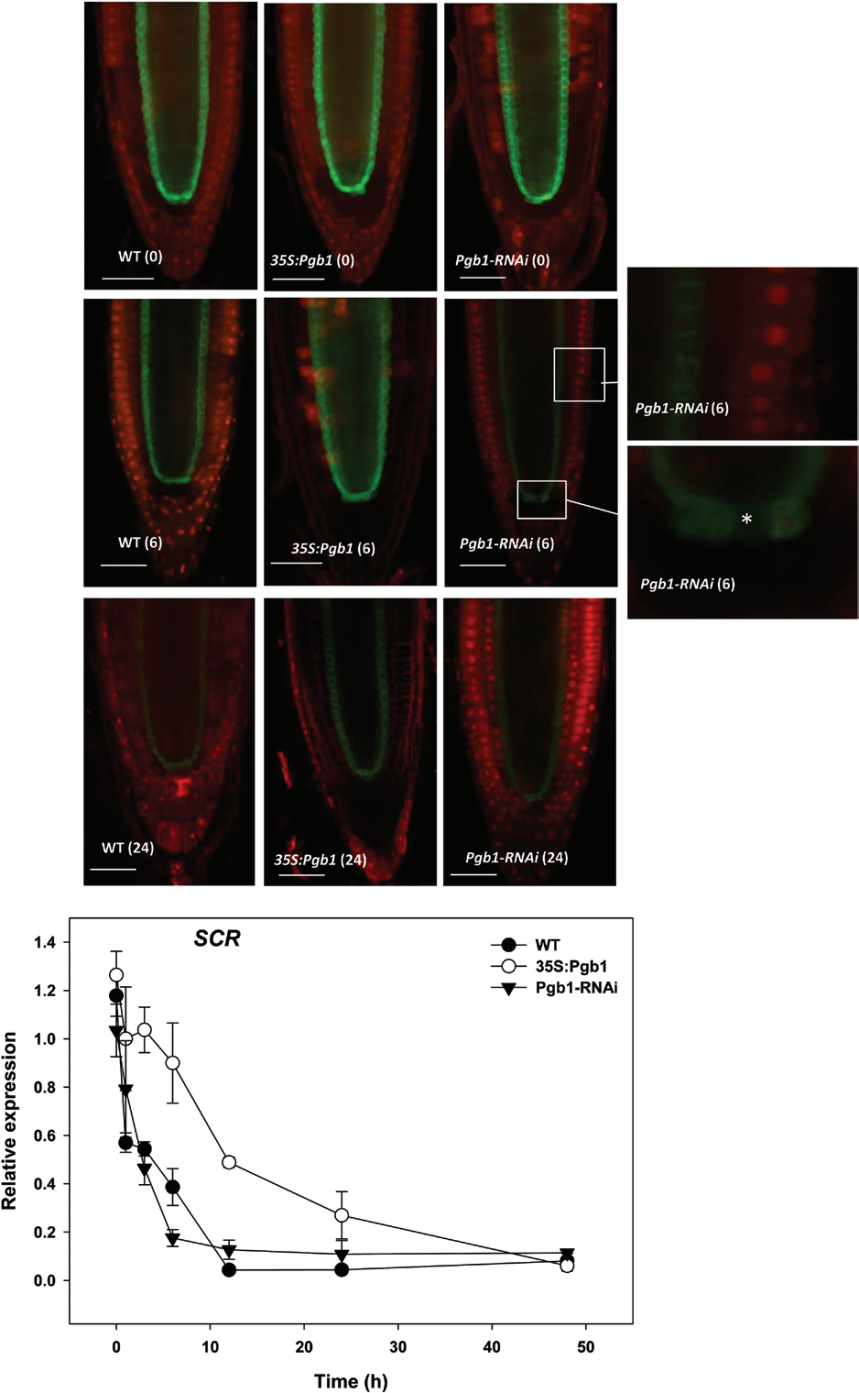
Localization and expression of SCARECROW (SCR) in the wild-type (WT) and lines overexpressing (35S:Pgb1) or down-regulating (Pgb1-RNAi) *AtPgb1* grown for 48 h in the absence or presence of 40% PEG (–1.76M Pa). Numbers in brackets on the images indicate hours of treatment in PEG. Confocal images of SCR:GFP marking the endodermis and quiescent cells (QCs), and the relative abundance of *SCR* transcripts; * indicates quiescent cell. The expression values are normalized to the WT at 0 h (set at 1), and are means ±SE of three biological replicates. Scale bars are 50 μm.

The signals of two other key root markers, SCR and WER, the first demarking the endodermis and QCs, and the second the lateral root cap (see Supplementary Fig. S1B), were also affected by water stress. The fluorescence signal and the transcript levels of SCR decreased gradually in WT roots treated with PEG (Fig. 7). This decrease was less pronounced in roots overexpressing *AtPgb1*, while in those suppressing *AtPgb1* the signal was very weak after only 6 h in PEG (Fig. 7). QCs were the first to lose the GFP fluorescence (asterisk in Fig. 7).

Application of PEG to WT roots resulted in a decline of *WER* transcripts and a concomitant loss of fluorescent signal, starting from the most mature domains of the lateral root cap (see Supplementary Fig. S8). After 24 h in PEG, the *WER* signal was very faint and only visible within the very innermost lateral root cap cells. A rapid decline in *WER* fluorescence was observed in roots suppressing *AtPgb1* after only 4 h in PEG, with no signal detected in these roots after 12 h. This was in contrast to roots overexpressing *AtPgb1*, which exhibited the highest WER signal throughout the imposition of water stress (see Supplementary Fig. S8).

Collectively, these results demonstrate that PEG stress compromises cell fate specification and tissue patterning in the RAM, and that these effects are aggravated by suppression of *AtPgb1* and alleviated in those situations where *AtPgb1* is induced. Notably, suppression of *AtPgb1* alters the functionality of the WOX5-expressing QCs, leading to the premature differentiation of the stem cells and a reduction in RAM size.

### Auxin gradient at the root tip is influenced by *AtPgb1*

Proper functioning of the QCs is ensured by the basipetal PIN-mediated flow of auxin, which accumulates at the centre of the RAM (Vieten *et al.*, 2005). Dissipation of auxin maxima at the root tip or interference with auxin translocation alters the behaviour of the RAM (Lee *et al.*, 2013). Based on the premise that NO perturbs the PIN-mediated movement of auxin, producing a phenocopy of the RAM abnormalities described in the present study (Fernandez-Marcos *et al.*, 2011), and that AtPgb1 is an effective NO scavenger (Hebelstrup *et al.*, 2006), auxin flow and accumulation were monitored in PEG-treated roots with altered levels of *AtPgb1*. The pattern of auxin flow was estimated by analysing the expression and localization of PIN1 (using a PIN1-GFP construct), the auxin efflux factor responsible for the basipetal translocation of auxin in the stele and endodermis (Vieten *et al.*, 2005), and PIN4 (using the PIN4:GUS construct), which regulates auxin movement and redistribution at the root tip (Friml *et al.*, 2002) (see Supplementary Fig. S1A). Auxin maxima were visualized by the activity of DR5:GUS (Ni *et al.*, 2001).

**Fig. 8.**
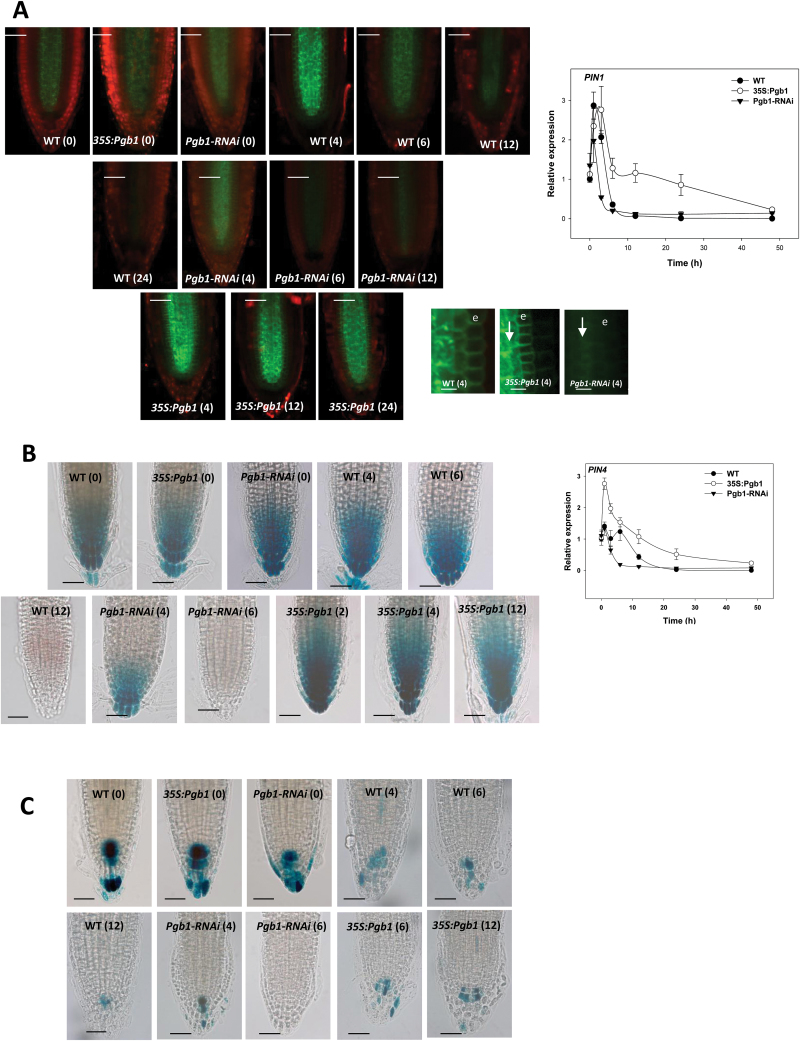
Auxin flow and accumulation during PEG-induced water stress in the wild-type (WT) and lines overexpressing (35S:Pgb1) or down-regulating (Pgb1-RNAi) *AtPgb1* grown for 48 h in the absence or presence of 40% PEG (–1.76M Pa). Numbers in brackets on the images indicate hours of treatment in PEG. (A) Confocal images of PIN1-GFP, and relative abundance of *PIN1* transcripts during the imposition of water stress. Arrows indicate the basipetal flow of auxin; e, endodermis. Scale bars are 50 μm (larger panels) and 5 μm (smaller panels). (B) Expression of PIN4:GUS and relative abundance of *PIN4* transcripts in roots. The relative expression values in (A) and (B) are normalized to the WT at 0 h (set at 1), and are means ±SE of three biological replicates. Scale bars in (B) are 50 μm. (C) Localization of DR5:GUS in roots of the different lines subjected to PEG-induced water stress. Scale bars are 50 μm.

The expression of *PIN1* was induced in all lines during the first hours in PEG and then it declined, especially in WT and *AtPgb1*-suppressing roots (Fig. 8A). Roots overexpressing *AtPgb1* retained the highest levels of *PIN1* transcripts. This expression pattern was confirmed by the localization signal which, relative to the WT, was very strong in the roots overexpressing *AtPgb1* throughout the course of the experiment. A sharp decline in PIN1-GFP fluorescence was observed in *AtPgb1*-suppressing roots after only 4 h in PEG, especially within the endodermis, the first tissue to lose the signal (‘e’ in Fig. 8A).

After an initial induction, most pronounced in the roots overexpressing *AtPgb1*, the levels of *PIN4* transcripts decreased during the imposition of PEG stress (Fig. 8B). During the first 24 h this decrease was inversely related to the amount of AtPgb1 present in the tissue. Localization studies confirmed this trend and showed that differences among lines occurred after 4 h in PEG when the *AtPgb1*-suppressing roots displayed a fainter GUS signal, which was completely lost after 6 h in PEG. This was in contrast to roots up-regulating *AtPgb1* in which a strong signal was retained even at 12 h in PEG, when no GUS was detected in the WT roots (Fig. 8B).

The auxin signal, visualized by the activity of DR5:GUS, was strong in WT roots at the beginning of the experiment (0 h in PEG), but dissipated gradually during water stress (Fig. 8C). After 12 h only a few cells in the centre of the RAM accumulated auxin. Relative to the WT, the GUS domain in the *AtPgb1*-suppressing roots was reduced after only 4 h in PEG, and was completely lost during the following two hours. A significant number of GUS-stained cells were still observed in the centre of the RAM of roots up-regulating *AtPgb1,* even after several hours of water stress (Fig. 8C).

Taken together, these findings suggest that PEG stress alters the PIN-mediated accumulation of auxin at the root tip, and they demonstrate that the presence of AtPgb1 is required to attenuate these changes. A rapid decline in PIN1 and PIN4 occurs in *AtPgb1* down-regulating roots after only a few hours of water deficit and this is accompanied by the dissipation of the auxin maxima within the RAM.

## Discussion

Short or extended periods of severe water deficit can compromise plant growth and induce death of root cells (Ramachandra Reddy *et al.*, 2004; Duan *et al.*, 2010). Through the use of PEG, it has been previously demonstrated that the deleterious effects of drought on root growth are the result of premature differentiation of the RAM (Ji *et al.*, 2014), and extensive death at the root tip (Duan *et al.*, 2010). The recurrence of these events during several types of stress reveals the susceptibility of roots to adverse environmental conditions. It has been suggested that Pgbs might exercise a protective role that allows roots to cope with diverse types of stress (Mira *et al.*, 2016*b*). For example, the hypoxia-inhibition of root growth resulting from differentiation and death of root cells was attenuated by the overexpression of *Pgb*s and aggravated when *Pgb*s were down-regulated (Mira *et al.*, 2016*b*). Based on these observations, the present work examined the involvement of Pgbs on death processes in root cells triggered by lethal applications of PEG.

The Arabidopsis *AtPgb1*, rapidly induced in roots at the onset of water stress (Fig. 1B), influenced root responses to water deficit, with high levels (35S:Pgb1 line) alleviating the PEG-growth inhibition, and low levels (Pgb1-RNAi) accentuating the inhibition (Fig. 1A). The protective role of AtPgb1 during water deficit appears to be linked to the ability of the protein to reduce PCD at the root tip (Fig. 2), possibly by limiting ethylene synthesis (Table 1), ethylene response (estimated by the expression levels of *ERF1*, *2*, and *10*) (Fig. 4), and accumulation of ROS (Fig. 5), all of which are known effectors of the death program. The involvement of both ethylene and ROS in the AtPgb1 response was demonstrated pharmacologically, with 1-MCP, an inhibitor of ethylene perception (Takahashi *et al.*, 2015), and DPI, an inhibitor of NADPH-oxidase (Bindschedler *et al.*, 2006) partially rescuing growth in *AtPgb1*-suppressing roots, and ET, an ethylene-releasing agent (Zhang *et al.*, 2010*b*) suppressing growth in roots over-expressing *AtPgb1* (Fig. 3B). These phenotypes were associated with changes in PCD patterns as a result of the pharmacological treatments (see Supplementary Fig. S6).

Programmed cell death in plant cells can be triggered by many signals, some of which originate from the endoplasmic reticulum (ER) (Zuppini *et al.*, 2004; Watanabe and Lam, 2008). One of the key functions of the ER is to regulate protein maturation and maintain a proper equilibrium between unfolded and folded proteins. Alteration of this equilibrium, elevating the levels of unfolded or misfolded proteins, triggers transduction pathways culminating with apoptosis in animals and PCD in plants (Boyce and Yuan, 2006; Watanabe and Lam, 2008). Evidence from our current study supports the findings of Duan *et al.* (2010) that documented ER-induced PCD in water-stressed tissue, and further suggests that *AtPgb1* might reduce the death program by alleviating ER stress through the activation of the unfolded protein response (UPR). This UPR is highly conserved among species (Boyce and Yuan, 2006) and involves the participation of AtBI-1, AtBiP2, and AtPR1, attenuators of ER stress that were all induced in roots overexpressing *AtPgb1* and suppressed in roots down-regulating *AtPgb1* (Fig. 3A). Often referred to as ‘survival factor’, the ER-located AtBI-1 suppresses the BAX (pro-apoptotic protein) activation of cell death in mammalian cells (Huckelhoven, 2004). Overexpression of *AtBI-1* in plants alleviates ER stress and reduces PCD progression, while its suppression accelerates the death program (Watanabe and Lam, 2006, 2008). The AtPgb1 regulation of *AtBiP2* and *AtPR1* was consistent with that of *AtBI-1*. AtBiP2 is a HSP70-type of chaperon involved in the secretion of proteins in the ER and a marker of UPR induction in eukaryotes (Koizumi *et al.*, 2001), while AtPR1 is a pathogenesis-related protein and a downstream intermediate of ER signalling (Watanabe and Lam, 2008). The contribution of ER-stress to the death program was further confirmed by the ability of PBA, a chaperon reducing ER stress through the stabilization of protein conformation (Watanabe and Lam, 2008), to alleviate the PEG-induced retardation of growth (Fig. 3B) and to suppress PCD (see Supplementary Fig. S6).

Commencement of the death program is often preceded by cellular differentiation (Ramsdale, 2012), a premature event observed in water-stressed wheat roots (Ji *et al.*, 2014), that can alter cell fate and tissue patterning in the RAM (Shishkova *et al.*, 2008). In Arabidopsis, the RAM surrounds the QCs, comprising mitotically inactive cells with the unique function to maintain the proximally and laterally situated stem cells in an undifferentiated state (Dolan *et al.*, 1993 and Supplementary Fig. S1A). The delicate balance between the rate of proliferation of the stem cells and the differentiation of their derivatives, ensuring a constant meristem size, makes the RAM extremely vulnerable to environmental perturbations. Evidence presented in this paper suggests that during water deficit *AtPgb1* expression contributes to the persistence of a functional meristem through the specification of the WOX5-expressing QCs. The retention of *WOX5* expression observed in the *AtPgb1*-overexpressing roots subjected to PEG stress (Fig. 6B) ensures the proper function of the QCs as the ‘organizing centre’ of the RAM, thus preserving the size (Fig. 6A) and function of the RAM. In contrast, suppression of *AtPgb1* resulted in the premature loss of *WOX5* expression after only a few hours in PEG, causing the differentiation of columella stem cells that accumulate starch granules (Fig. 6C). The loss of pluripotency and differentiation of the stem cells is a plausible cause of the smaller RAM observed in the *AtPgb1*-suppressing roots (Fig. 6A). Through the specification of QC identity, WOX5 acts as a regulator of stem cell maintenance by repressing the differentiation factor CDF4 in the adjacent stem cells (Pi *et al.*, 2015). Consistent with our results, the ablation of the QCs (van den Berg *et al.*, 1997) or the loss of function associated with the *wox5* mutant (Sarkar *et al.*, 2007) cause the differentiation of the subtending columella initials, while ectopic *WOX5* expression is sufficient to reprogram fully differentiated columella cells into stem cells (Pi *et al.*, 2015). The rapid, PEG-induced loss of root tissue specification, aggravated by suppression of *AtPgb1* and delayed by its up-regulation, was also apparent through the localization of SCR (Fig. 7). In addition to participating in radial root patterning by controlling the asymmetric cell division pattern of the daughter of the cortex–endodermis initial (Di Laurenzio *et al.*, 1996), SCR is also required for proper QC activity (Sabatini *et al.*, 2003). The early (6 h in PEG) suppression of *SCR* in the *AtPgb1*-suppressing roots, especially in the QCs (asterisk in Fig. 7), reinforces the argument for the requirement of *AtPgb1* in delaying the loss of QC specification during water deficit. Together with cell specification and tissue patterning in the central domains of the RAM, it cannot be excluded that *AtPgb1* delays the loss of tissue identity in the periphery of the root tip, as evidenced by the changes in expression and localization patterns of the lateral root cap marker WER (see Supplementary Fig. S8). The pattern of cellular proliferation, estimated by the GUS activity of two cyclins (cycB3,1:GUS in the meristematic region and cycA1,3:GUS in mature tissue; see Supplementary Fig. S9), further confirms the requirement of *AtPgb1* for normal root growth under conditions of water deficit.

Maintenance and function of the RAM is controlled by the PIN-mediated accumulation of auxin at the root tip, and alterations in auxin flow by high levels of NO lead to the same meristem abnormalities observed in our study, namely a decrease in meristem size and premature differentiation of meristematic cells (Fernandez-Marcos *et al.*, 2011). This observation, in conjunction with the well-documented ability of *AtPgb1* to scavenge NO (Hebelstrup *et al.*, 2006), prompted us to determine whether the early meristematic defects observed in water-stressed *AtPgb1*-suppressing roots were associated with perturbations in the flow and accumulation of auxin in the RAM. The patterns of *PIN1* and *4* expression and localization were rapidly altered in tissue suppressing *AtPgb1* after only a few hours in PEG (Fig. 8A, B). These alterations, denoting changes in the long-range basipetal canalization of auxin mediated by PIN1 (Petersson *et al.*, 2009) and its maintenance and redistribution within the meristematic cells, regulated by PIN4 (Friml *et al.*, 2002), reflect the rapid (6 h in PEG) disappearance of auxin in the RAM (Fig. 8C). Abnormal root tips have been associated with defective PIN1 localization, while misexpression of PIN4 causes deviations in QC fate (Friml *et al.*, 2003). The retention of a PIN-directed auxin maxima at the RAM, and specifically in the QCs (Petersson *et al.*, 2009), is paramount for meristem functionality and, most importantly, for modulating cell fate regulators, including WOX5 (Ding and Friml, 2010) and SCR (Sabatini *et al.*, 1999). Mutants exhibiting decreasing DR5:GUS activity displayed aberrations in cell specification and tissue patterning (Sabatini *et al.*, 1999).

**Fig. 9.**
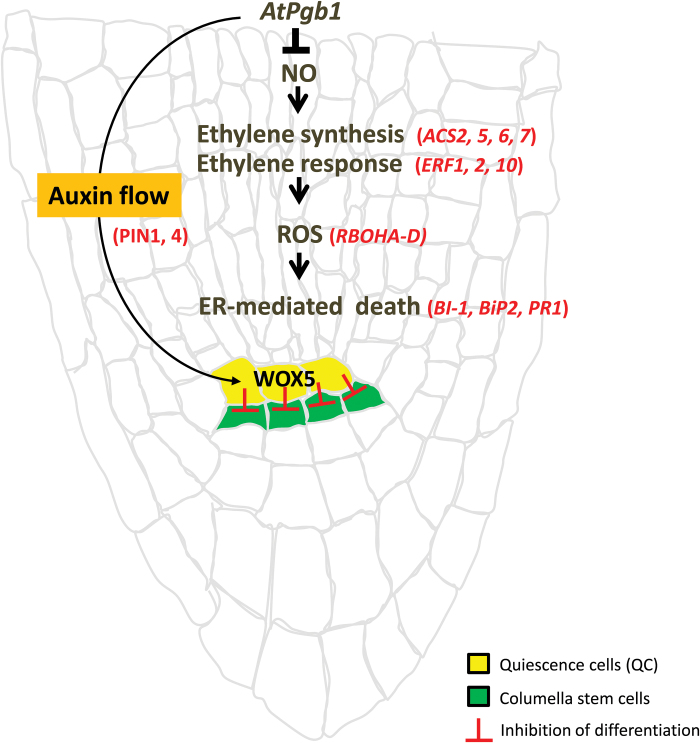
Proposed model of AtPgb1 action during severe water stress. The model is based on the current work and integrates some information from previous studies (Mira *et al.*, 2016*b*). By suppressing production of nitric oxide (NO), AtPgb1 reduces the promotive effect of ethylene and ROS on ER-mediated death of root cells. AtPgb1 is also required to maintain the fate of the QCs, which prevent the differentiation of the subtending columella stem cells. This effect, possibly mediated by the flow and distribution of auxin, contributes to the retention of a functional RAM during conditions of PEG stress. Genes whose activities have been measured are listed in red.

In conclusion, while not producing any visible root phenotype in the absence of PEG, the expression level of *AtPgb1* influences the root response to severe PEG stress. By modulating ethylene and ROS, AtPgb1 alleviates ER stress-induced death in PEG-stressed roots and delays the degradation of the RAMs by maintaining meristem functionality and fate identity in the WOX-5-expressing QCs. These events are most likely associated with the retention of the flow of auxin and its accumulation at the root tip (Fig. 9). The similarity of regulatory components shared by the root and shoot apical meristems, where the WOX-5 like WUSCHEL gene possesses stem cell-promoting functions in the shoot, as well as the presence of AtPgb1 in the shoot tip, suggest a potential role of this protein in protecting meristematic cells during conditions of extreme stress.

## Supplementary Data

Supplementary data are available at *JXB* online.

Fig. 1. Tissue patterning in the Arabidopsis root apical meristem.

Fig. 2. Water potentials of agar media infiltrated with PEG.

Fig. 3. Water stress inhibition of root growth.

Fig. 4. Time course of root elongation on different media.

Fig. 5. Expression of *AtPgb1* in wild-type roots grown with different water potentials.

Fig. 6. PCD in root tips of PEG-stressed roots.

Fig. 7. Alteration of expression of ethylene-biosynthetic genes by phytoglobin.

Fig. 8. WER expression and localization.

Fig. 9. Cell division patterns in PEG-treated roots.

Table S1. List of primers used in this study.

## Supplementary Material

Supplementary_Figures
