# Supplementary material for: Expression of Arabidopsis class 1 phytoglobin (AtPgb1) delays death and degradation of the root apical meristem during severe PEG-induced water deficit
Source: J Exp Bot. 2017 Oct 20;68(20):5653–68. doi: 10.1093/jxb/erx371 (PMC5853930; doi:10.1093/jxb/erx371)
Supplement: Supplementary_Figures [file erx371_suppl_supplementary_figures.pdf]

Expression of the Arabidopsis class 1 Phytoglobin (*AtPgb1*) delays death and degradation of the root apical meristem (RAM) during severe PEG-induced water deficit

Mohamed M. Mira[1], Shuanglong Huang[2], Karuna Kapoor[2], Cassandra Hammond[2], Robert D. Hill[2], and Claudio Stasolla[2]*

Supplemental files

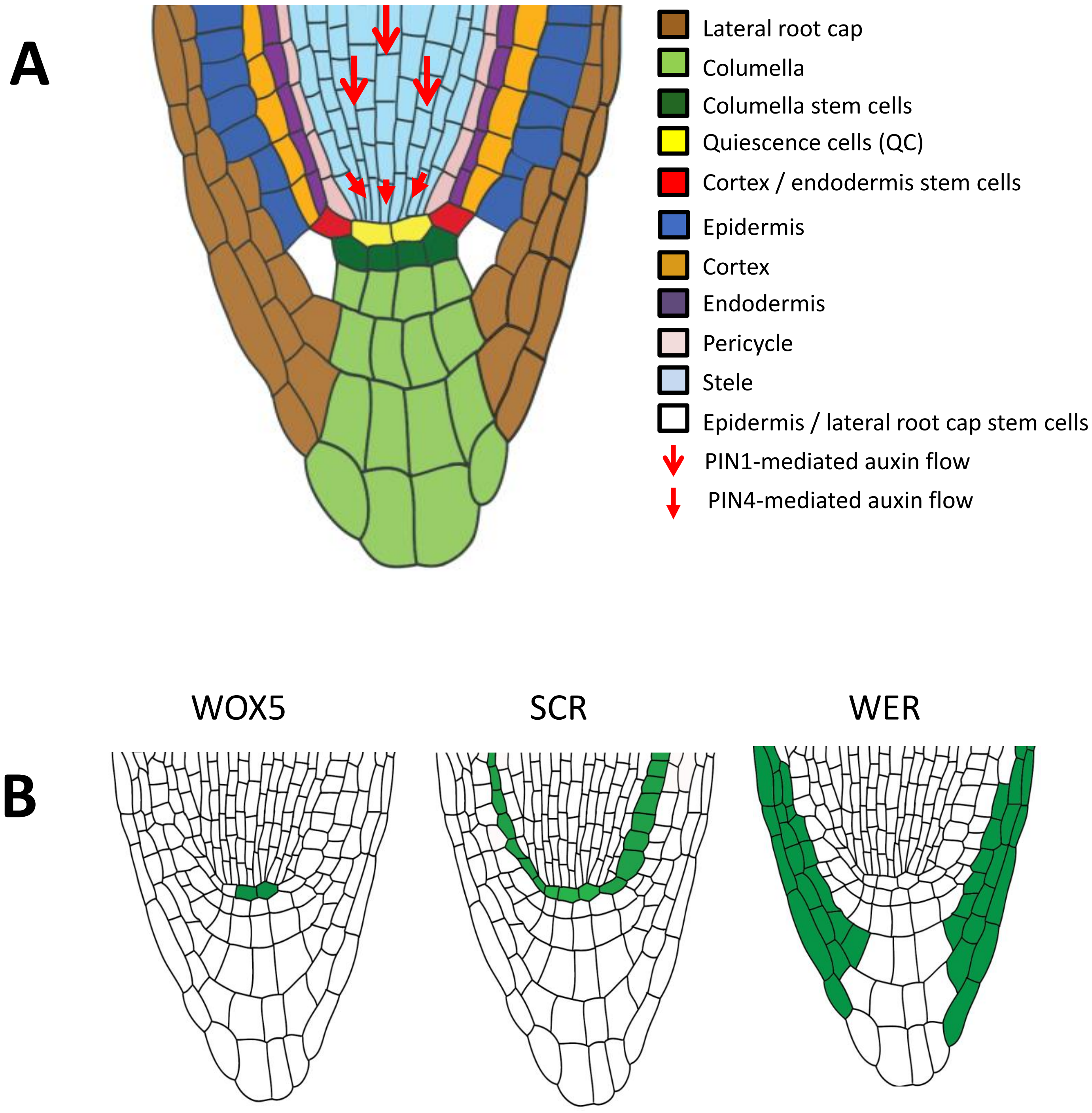

Supplemental Fig. 1. Tissue patterning in the Arabidopsis root apical meristem. **(A)** Root morphology. **(B)** Expression domains of WOX5, marker of the QC; SCR, marker of the endodermis and QC; and WER, marker of the lateral root cap.

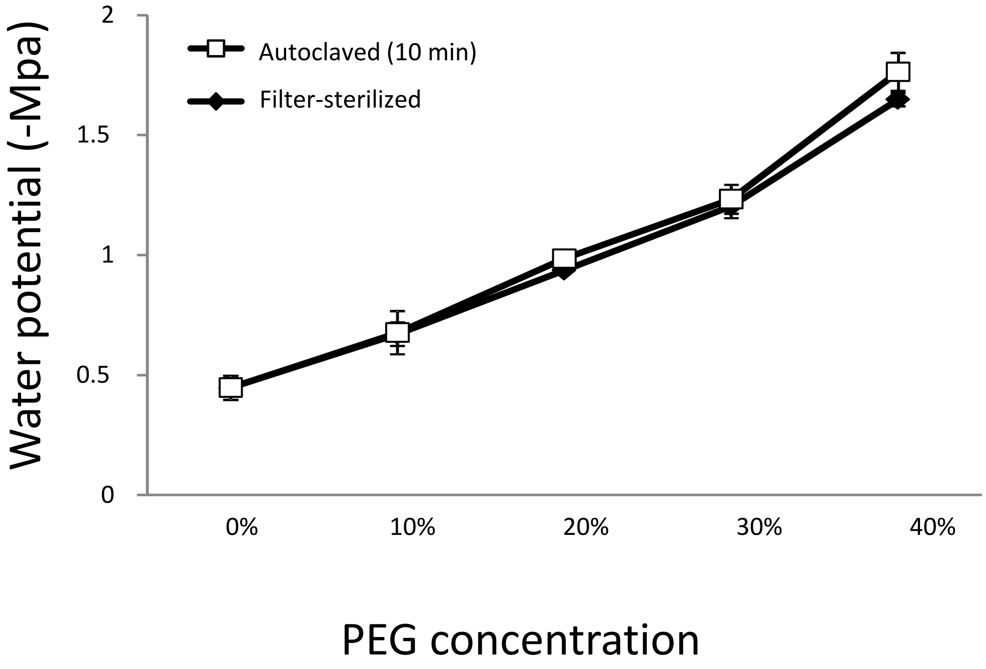

Supplemental Fig. 2. Water potentials (-MPa) of agar media infiltrated with autoclaved or filter-sterilized PEG. Values ± SE are means of readings from four plates.

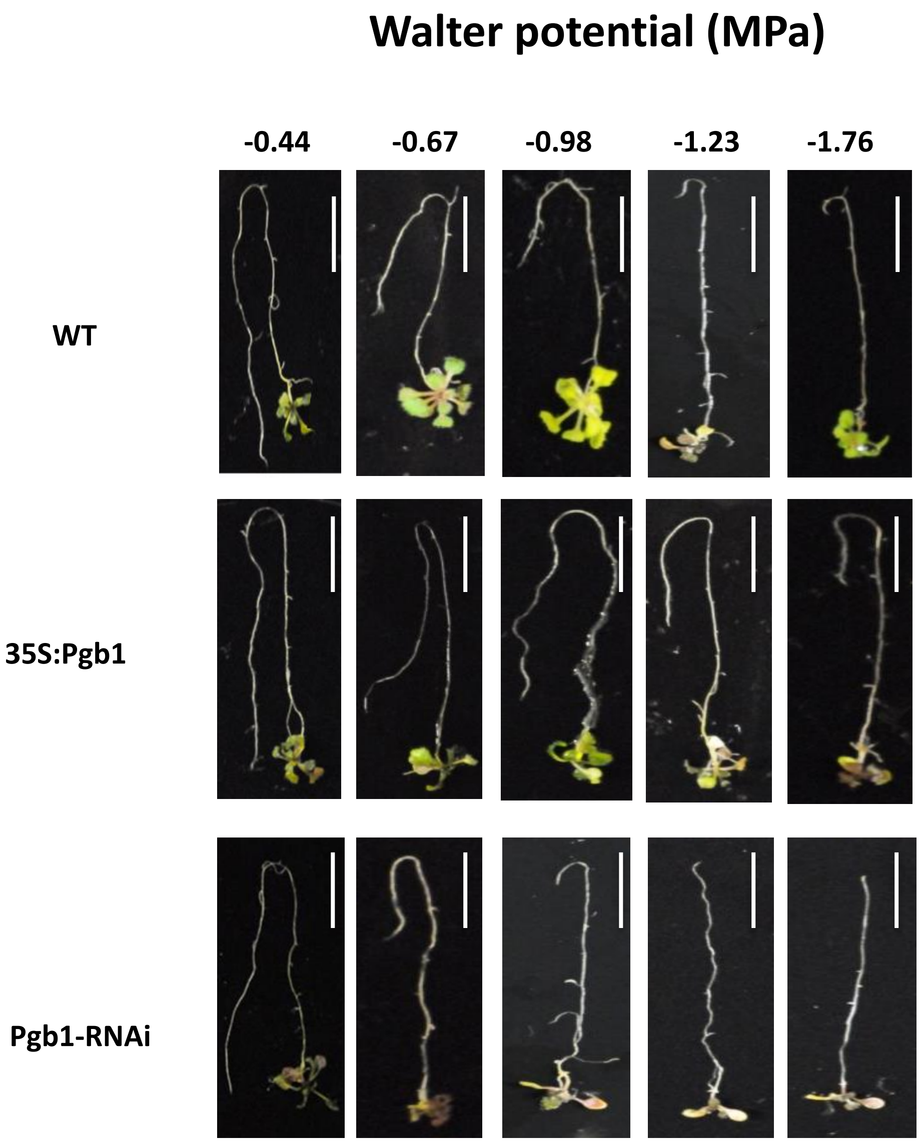

Supplemental Fig. 3. Water stress inhibits root growth. Root growth of four-day old Arabidopsis seedlings cultured for 6 days on PEG-containing medium. Seedlings were rotated by 180 degree when transferred on PEG to show new root growth. Scale bars = 5 mm.

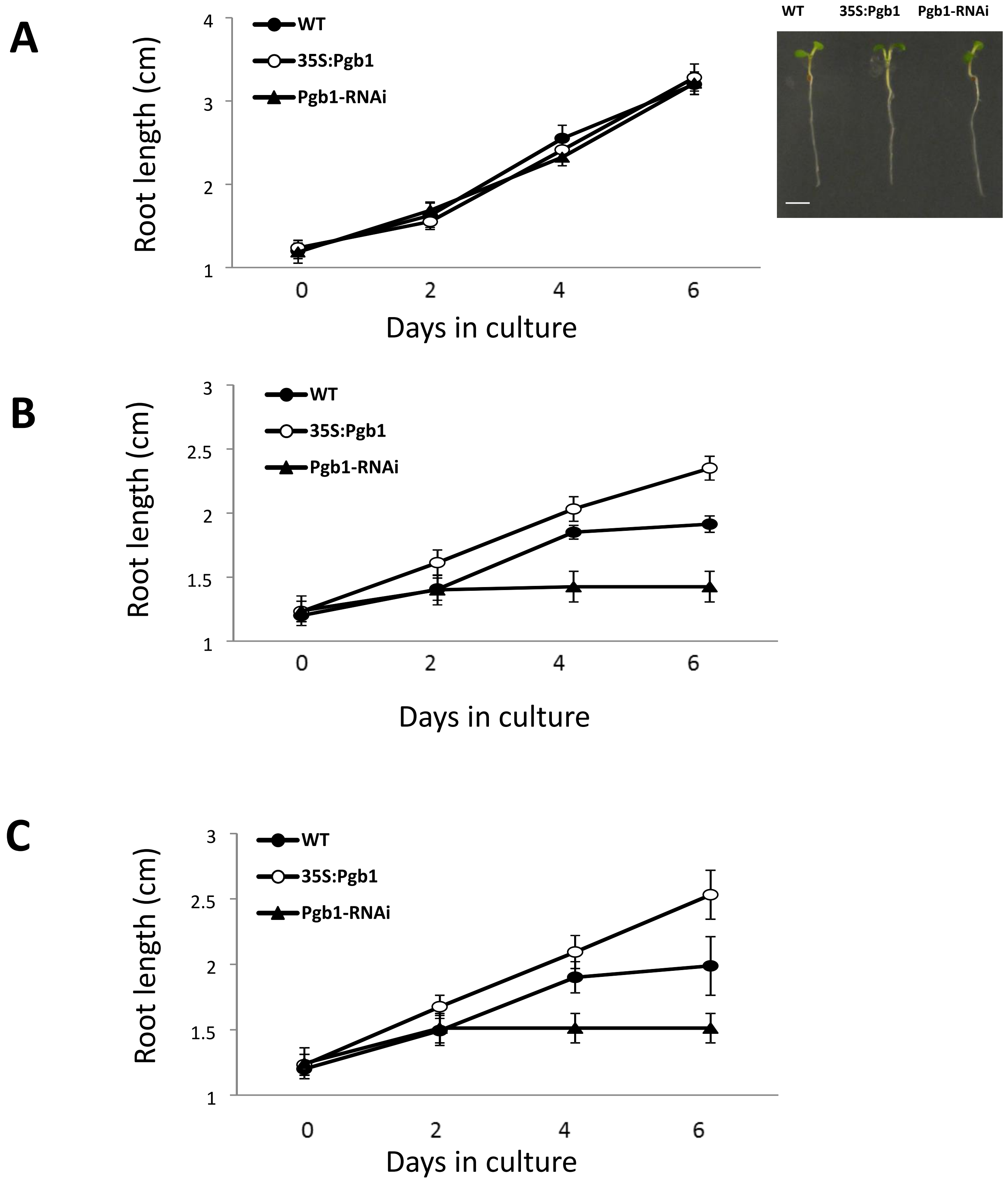

Supplemental Fig. 4. Time course of root elongation on media devoid of PEG **(A)**, and media containing 40% PEG autoclaved for 10 min **(B)** or filter sterilized **(C).** The phenotype of seedlings of the three lines at the beginning of the experiment (day 0) is also shown. Scale bar = 2.5 mm.

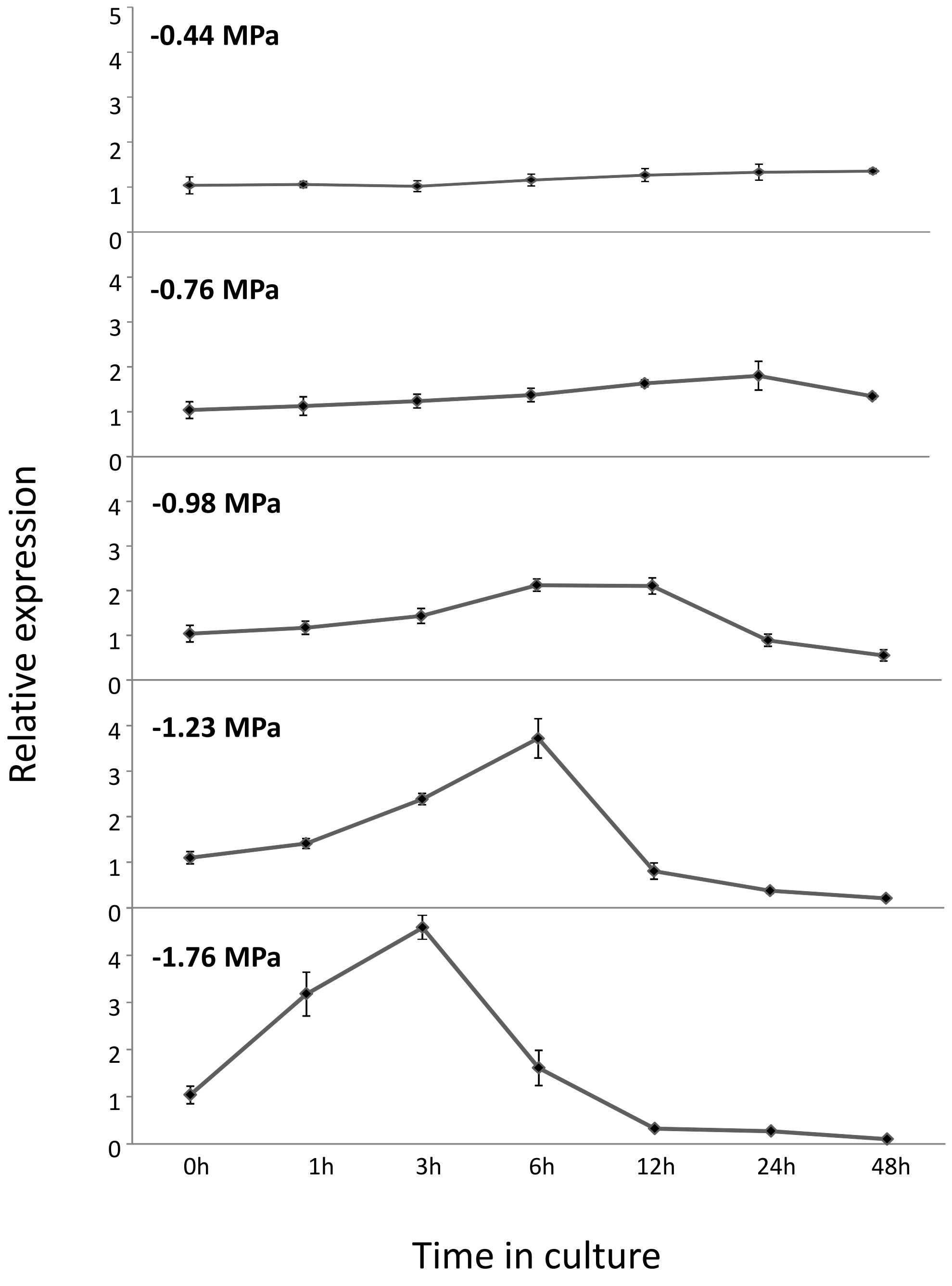

Supplemental Fig. 5. Expression of *AtPgb1* in wild type roots grown with different water potential. Expression values are means ± SE of three biological replicates and normalized to the value of 0h set at 1

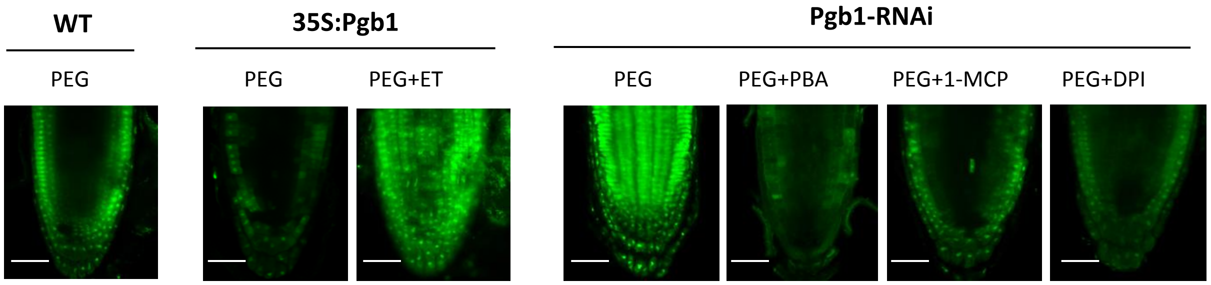

Supplemental Fig. 6. PCD in root tips of PEG stressed roots. Root growth of Arabidopsis seedlings cultured for 48h on 40% PEG (-1.76 Mpa). ET, ethephon; 1-MCP, 1-methylcyclopropene; PBA, 4-phenyl butyric acid; DPI, diphenyleneiodonium. Scale bar = 50 μm.

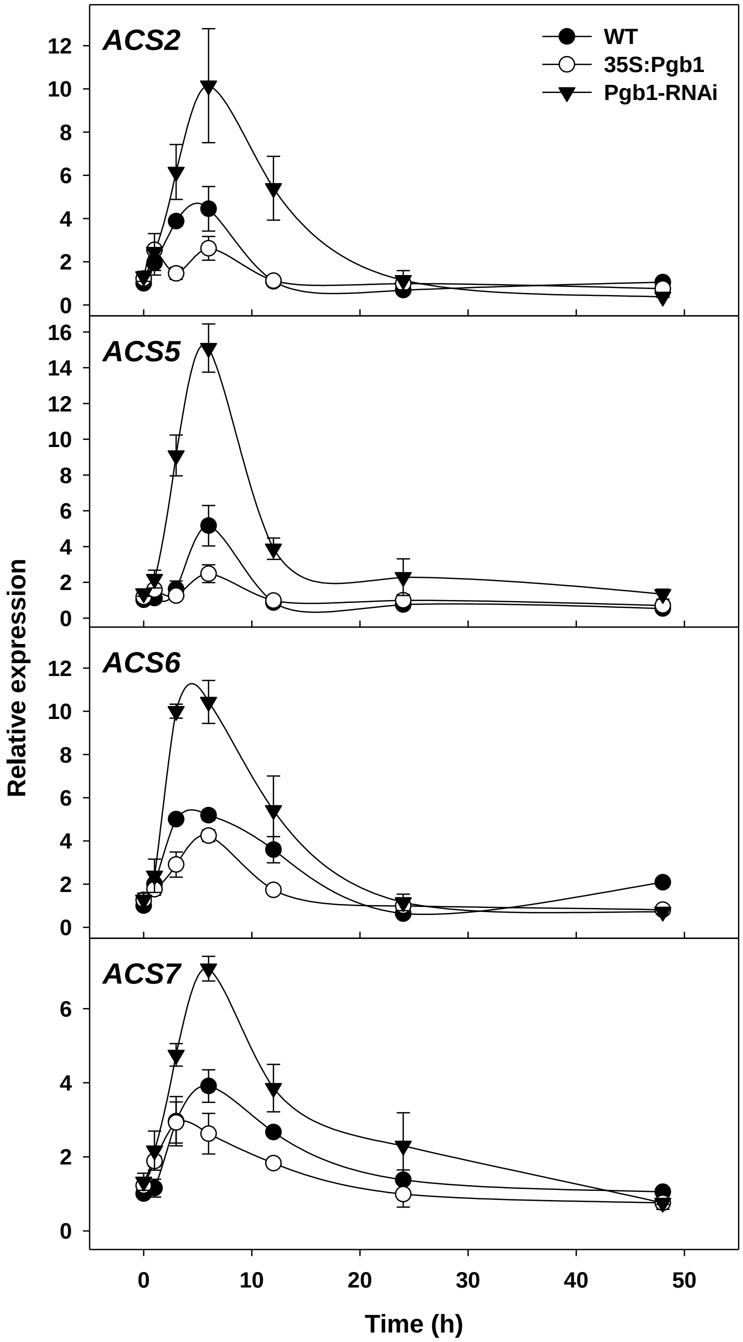

Supplemental Fig. 7. Phytoglobin alters the expression of ethylene biosynthetic genes. Relative expression level of *Aminocyclopropane-1-carboxylic acid synthase* (*ACS*) *2, 5, 6,* and *7* in roots of the WT line and lines over-expressing (35S:Pgb1) or down-regulating (Pgb1-RNAi) *AtPgb1*.
Roots were grown on 40% PEG (-1.76MPa) for 48h. Values, normalized to the WT (0h) value set at 1, are means ± SE of three biological replicates.

A

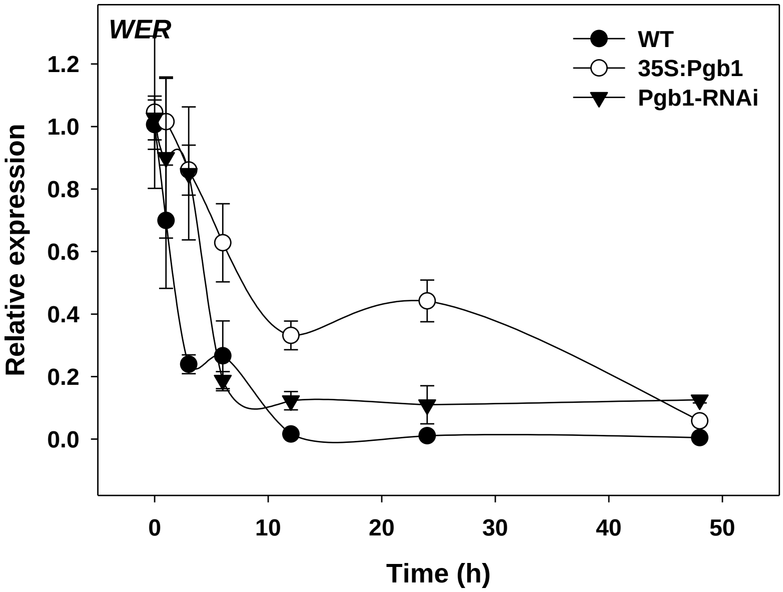

B

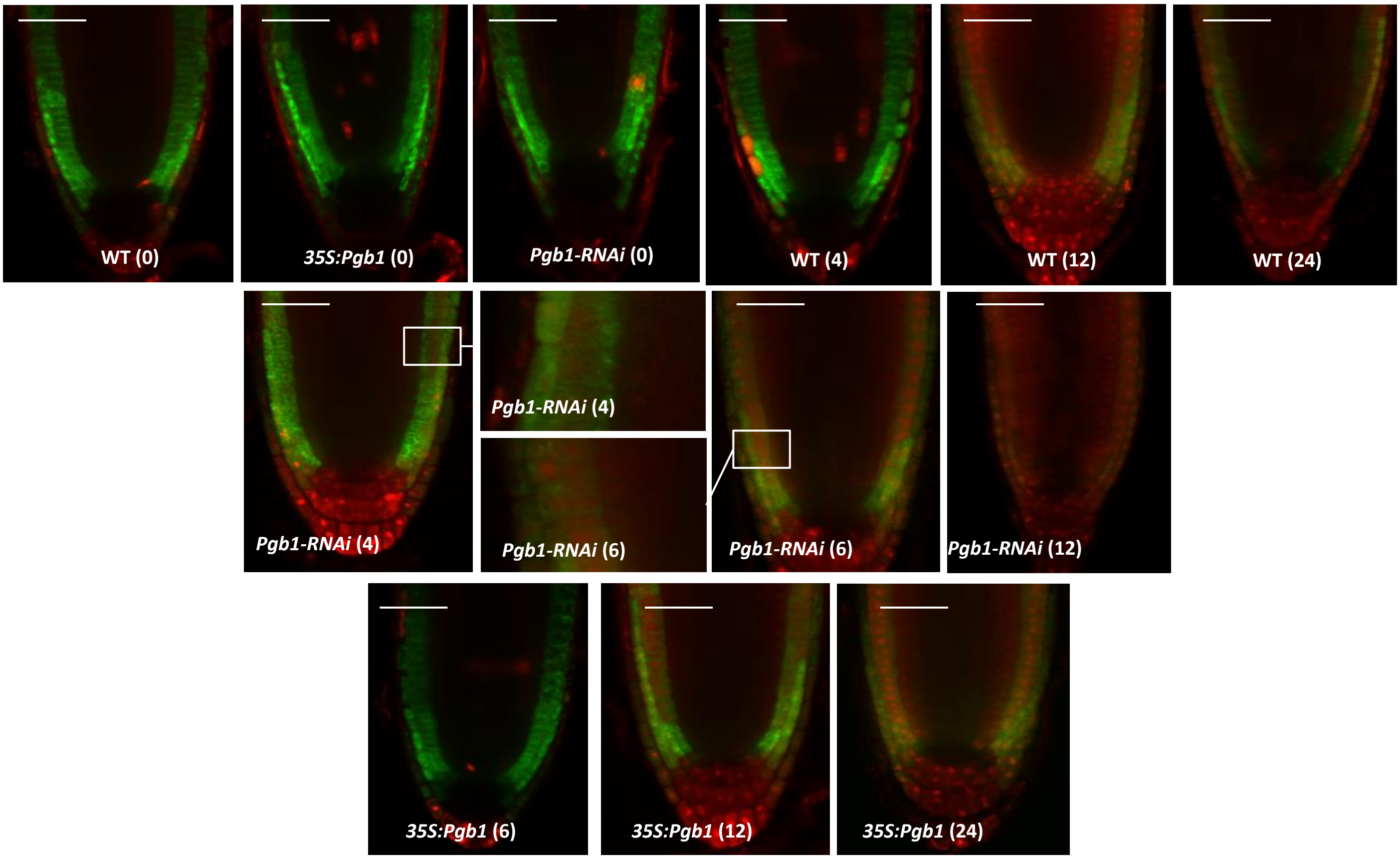

Supplemental Fig. 8. WER expression and localization. **(A)** Changes in *WER* expression in the presence of 40% PEG (-1.76MPa) over time. The relative expression values, normalized to the WT(0h) value set at 1, are means + SE of three biological replicates. **(B)** Confocal images of pWER:GFP marking the lateral root cap. Number in brackets indicates hours in PEG. Scale bars = 50 μm.

A

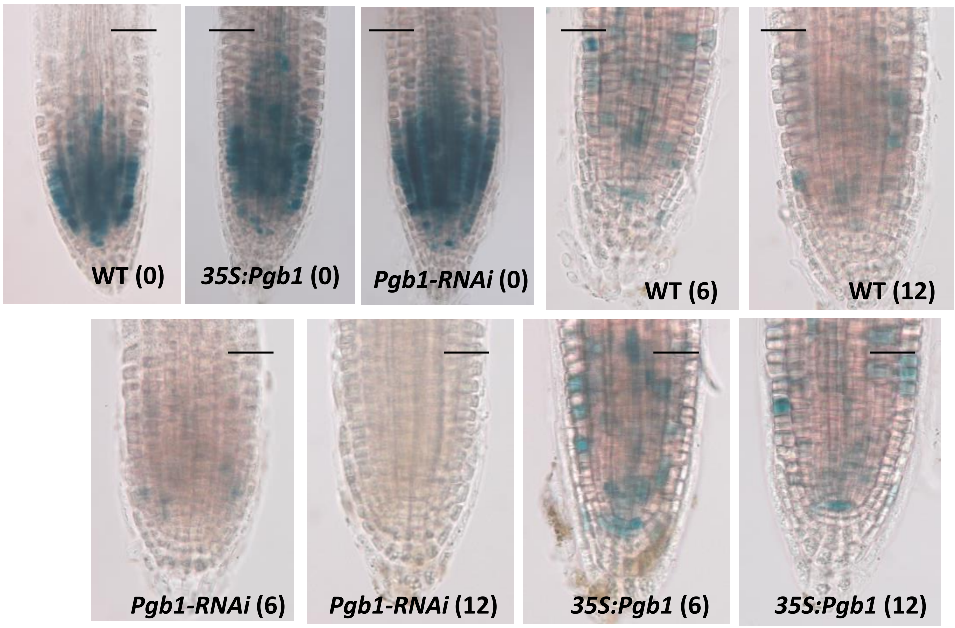

B

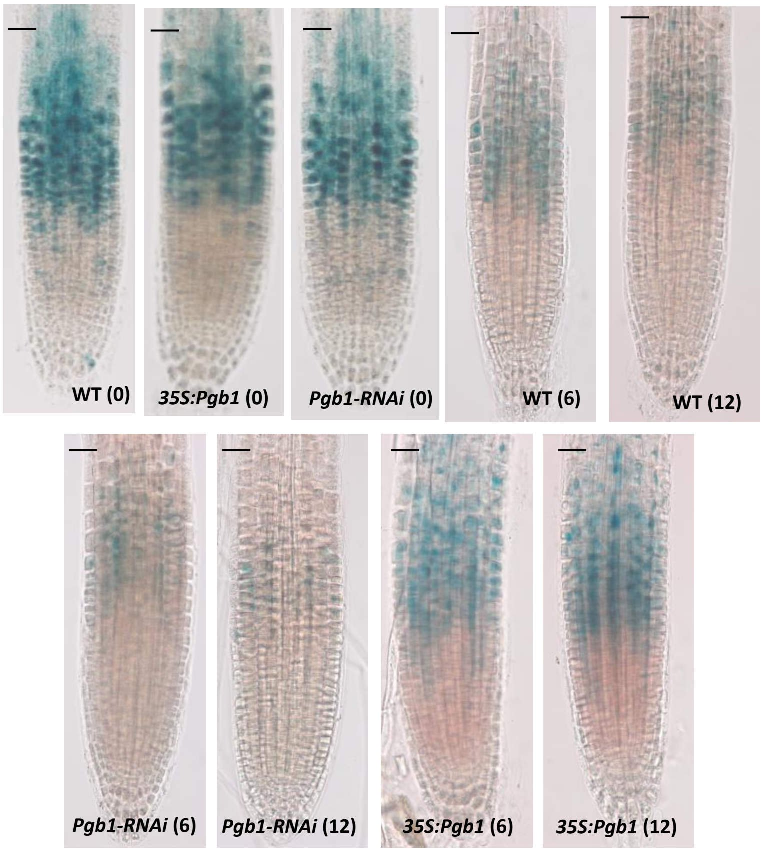

Supplemental Fig. 9. Cell division pattern in PEG-treated roots. Localization of cyclin B3,1 using cycB3,1:GUS **(A)** and cyclin A1,3 using cyc A1,3:GUS **(B)** in root tips of Arabidopsis plants cultured in 40% PEG (-1.76MPa). Numbers in bracket indicate hours in PEG. Scale bars = 50 μm.

Supplemental Table 1. List of primers used

RBOHA	AGAACGTGATGGACGAAGTG	GAGACTGGAGCATCGTGATAAG
RBOHB	GTAAGTCGTTCGGTGCTATGT	CAAACCTCATTGCAACCTCATC
RBOHC	CAAGGAACAAGCCCAACTAAAC	CTCACGCGTAACCCAATAGAA
RBOHD	ACACCATCACTTGGCTTAGG	CGACTATCCCGCTTGCAATA
ETR1	GCTAGACGAGAAGCAGAAACA	GGAGTAAGGAAGAGAGTGCAATAA
ETR2	CCTCTGGATTCGATTGCCTTAC	CCACTTCATAACCGTCCATCTC
ERS2	GCTGACTGGTATCGTCTCTTTC	GCTCTCTGGTCTTCTTACTCAAC
At-ERF1	CCGCTCCGTGAAGTTAGATAAT	TCTTTCACCAAGTCCCACTATTT
Erf2	AAGTCGAGCCAACTGAGAAC	TCTGCCTCACTCCTCTGTAA
ERF10	CGAGTTTGTCCTGACCAGTTT	GGTTCCATTCGCAGCTTACA
AtACS2	GCGACTAACAATCAACACGGA	AGCAAAGCTGATTCTCTGCAA
AtACS5	CCTACTCCTTACTATCCTGGATTTG	TTGCAGAGCTGATTCCGTGA
AtACS6	TCAGACAAGCTGTAGCGAAA	AGAAAGCAACCGTCTCGTGT
AtACS7	TTTCAGACACTCACGGCGAA	ACGAGACCTGATTCTCAGCG
AtBI1	CTCTTGTGGCGTCTGCCTTT	CGTTGTAAGAATACCGCCGATAT
BiP2	GAGAAGATCGACGCCAGGAAT	TCGCTCACTTGGTTCTTCATGT
PR1	GTCTCCGCCGTGAACATGT	CGTGTTCGCAGCGTAGTTGT
PIN1	GAAGGATCCAGAAGGGAAGATTAT	GCAGAGCTACAGTCAGAAACA
PIN4	CCAAACACGTACTCCAGTCTAAT	CCATTCCAAGACCAGCATCT
WOX5	GTCAAGAGGAAGAGAAGGTGATAG	CTGGTTATTGCCTCTAGCTCTC
SCARECROW1	TTGTGGCTTCTCTGGTCTTC	AATGGCGTCAACCCATACA
WER	CCCGAATCCTACCGAAACATC	ACTCATCCTCATGAACCCAAAG
